# Comprehensive genomic profiling of mucosal melanoma reveals novel fusion transcripts and dysregulation of cell‐cycle, MAPK, and PI3K pathways

**DOI:** 10.1002/path.70108

**Published:** 2026-08-31

**Authors:** Matilde Monti, Martina Brigida Romanello, Luca Agnelli, Matteo Orlandi, Luca Bertoli, Sara Picinoli, Irene Pezzali, Giulio Eugenio Mandelli, Daniela Medicina, Giovanni Ribaudo, Adele Busico, Elena Conca, Vilma Pellegrini, Piera Balzarini, Daniele Gallucci, Anna Di Matteo, Davide Lombardi, Claudia Ghigna, Giancarlo Pruneri, Alessandra Gianoncelli, Marzia Rossato, William Vermi

**Affiliations:** ^1^ Department of Molecular and Translational Medicine University of Brescia Brescia Italy; ^2^ Department of Diagnostic Innovation, Pathology Unit 2 Fondazione IRCCS Istituto Nazionale Dei Tumori Milan Italy; ^3^ Department of Biotechnology University of Verona Verona Italy; ^4^ Unit of Pathology, Department of Molecular and Translational Medicine University of Brescia – ‘ASST Spedali Civili di Brescia’ Brescia Italy; ^5^ Istituto di Genetica Molecolare ‘Luigi Luca Cavalli‐Sforza’ Consiglio Nazionale delle Ricerche Pavia Italy; ^6^ Unit of Otorhinolaryngology – Head and Neck Surgery, Department of Medical and Surgical Specialties, Radiological Sciences and Public Health University of Brescia Brescia Italy; ^7^ Department of Pathology and Immunology Washington University School of Medicine St. Louis MO USA

**Keywords:** mucosal melanomas, sinonasal melanoma, genomic landscape, structural variants, fusion transcripts, single‐nucleotide variants, MAPK pathway, trametinib

## Abstract

Mucosal melanomas (MMs) are rare, aggressive cancers with poor outcomes and limited response to standard therapies. A significant knowledge gap exists regarding their genomic landscape and corresponding druggable targets. This study explores this issue through a multi‐omic analysis, including whole exome‐, RNA‐, and targeted sequencing, of unique sinonasal mucosal melanoma (SN‐MM) cell lines. SN‐MM cell lines showed a low tumour mutational burden and low genomic instability, suggesting that their DNA repair mechanisms are largely intact. However, they exhibited a high prevalence of structural variants, likely from catastrophic genomic events. We also found novel, clinically actionable variants, including oncogenic fusion transcripts. Key findings, such as fusion transcripts and single‐nucleotide variants, were validated on diagnostic biopsies. Moreover, using a break‐apart FISH assay, we identified *BRAF* rearrangements as recurrent events in MM, supporting their biological relevance. The MAPK pathway was found to have a significant functional role, sustained by variants such as *BRAF* p.Asn581Ser, *NRAS* p.Gly12Asp, and the *MKRN1*::*BRAF* fusion. This pathway activation was effectively inhibited by the MEK inhibitor trametinib. This genomic and functional profiling unveiled novel molecular hubs in MM. The findings propose that MEK inhibitors may represent a relevant therapeutic option for *BRAF* fusions and non‐V600 *BRAF* mutant tumours, providing a novel strategy for this challenging disease. Further molecular and functional validation of novel variants, and particularly of *BRAF* rearrangements, may reveal additional clinically relevant therapeutic targets. © 2026 The Author(s). *The Journal of Pathology* published by John Wiley & Sons Ltd on behalf of The Pathological Society of Great Britain and Ireland.

## Introduction

Mucosal melanoma (MM) is a highly aggressive and rare form of melanoma arising from melanocytes in mucosal membranes at various anatomical sites [1]. Head and neck tumours, particularly those arising in the sinonasal (SN) cavity, represent the most common MMs. The majority of patients undergo surgical resection, often followed by adjuvant radiotherapy [2], but local recurrence and distant spread are common and result in poor outcome, with a 5‐year overall survival rate of less than 30% [3, 4]. Adjuvant systemic chemotherapies do not provide meaningful survival benefits, with most patients dying early after the diagnosis due to disease dissemination [5]. Immune checkpoint blockade (ICB) and immunomodulatory therapies confer a survival benefit to MM patients with metastatic disease; however, the response rates remain significantly lower than those in cutaneous melanoma (CM) [6, 7]. Compared with CM, where recurrent molecular drivers have been established [8], the molecular basis of MM transformation and progression is poorly understood. The genomic MM landscape has been explored in small cohorts with focused targeted strategies, revealing that MM is mainly characterized by structural variation (SV) [9, 10, 11]. Moreover, the tumour mutational burden (TMB) is substantially lower in MM than in CM [9, 10]. Mitogen‐activated protein kinase (MAPK) is key in the development of the large majority of CMs, supported by the high frequency of *BRAF* p.Val600 and *NRAS* mutations. On the contrary, *BRAF* p.Val 600 mutations are found in a negligible percentage of MM patients, who instead tend to harbour atypical mutations in the *BRAF* gene [12, 13]. Sinonasal MMs (SN‐MMs) are commonly driven by *NRAS* substitutions (hotspots in p.Gly12, p.Gly13). *BRAF/RAS* wild‐type tumours display a complex landscape involving alterations in the RAS–MAPK, PI3K, and WNT pathways, including mutually exclusive loss‐of‐function (LoF) mutations in *NF1* and *SPRED1*, *SF3B1* mutations, and alterations in the *PIK3CA*, *PTEN*, and mTOR pathway [14, 15, 16, 17, 18, 19]. Moreover, SN‐MMs are characterized by amplification of oncogenes, including frequent *MYC* and *CDK4* amplifications, or deletion of tumour suppressor genes [14, 20]. Finally, many cases showed expression of pAkt and pErk, suggesting the activation of both PI3K/Akt and RAS/MAPK pathways [13, 21]. Unfortunately, this set of data, although of potential relevance, offers limited support to decision making for the clinical management of MMs. As for other rare cancers, extensive molecular profiling may suggest drug repurposing, or potential novel targets.

By exploiting well‐characterized MM cell lines [21] using genome‐wide sequencing strategies, this study expands our knowledge of the MM landscape. Of relevance, we uncovered novel clinically actionable variants, including oncogenic fusion transcripts and SNVs, and defined a significant functional role for the MAPK pathway in MM that may be blocked by administration of trametinib.

## Materials and methods

### Ethics approval and consent to participate

This study was approved by the Institutional Ethics Board of ASST Spedali Civili of Brescia (IRB code: NP 2066/2015) and ASST Sette Laghi of Varese (IRB code: NP 33025/2015), Italy. All patients who provided clinical specimens provided written informed consent.

### Human samples and cell culture

Five human SN‐MM cell lines, previously generated from tumour biopsies [21], and the corresponding formalin‐fixed paraffin‐embedded (FFPE) tumour tissues were used in this study, as detailed in the Supplementary materials and methods. Cell lines were treated with vemurafenib (10 mm; Cat. No. HY‐12057; MedChemExpress, Monmouth Junction, NJ, USA) or trametinib (10 mm; Cat. No. HY‐10999, MedChemExpress).

### Illumina TruSight Oncology 500 (TSO500) HRD assay

The SN‐MM cell lines were analysed using the commercially available TruSight Oncology 500 HRD assay (Illumina, San Diego, CA, USA) and sequencing was performed on the NextSeq 500 sequencer (Illumina), as detailed in the Supplementary materials and methods. DNA variant filtering and annotation were performed using the DRAGEN TruSight Oncology 500 Analysis Software v2.6.1 (Illumina). A custom variant filter was implemented using the OpenCRAVAT tool (v2.11.1; https://opencravat.org; last accessed 3 July 2025) to include only non‐synonymous variants with a population allele frequency less than 1 × 10^−4^, unless variants of uncertain significance (VUS) or not available (NA) in the ClinVar database were classified as pathogenic and Tier I or II according to VarSome (v13.2.0; https://varsome.com; last accessed 3 July 2025) clinical databases. Variants classified as benign or likely benign in ClinVar were excluded. Common single nucleotide polymorphisms (SNPs) were filtered out using dbSNP (https://www.ncbi.nlm.nih.gov/snp/; last accessed 3 July 2025) and 1000 Genomes (https://www.internationalgenome.org/home/; last accessed 3 July 2025) databases. The cut‐off for variant allele frequency (VAF) was set at 5%. The TSO500 assay estimates TMB by calculating the number of eligible passing variants and dividing it by the size of the targeted coding region covered by the panel. A TMB value greater than ten mutations per megabase (Mb) was classified as ‘high’ (≥ 10 mut/Mb).

### Whole exome sequencing (WES)

WES libraries were prepared from 50 ng of genomic DNA using the Twist Enzymatic Fragmentation (EF) Library Preparation Kit (Twist Bioscience, South San Francisco, CA, USA), with universal adapters, followed by target enrichment using the Twist Human Core Exome panel (version 1.3) following the manufacturer's instructions. WES libraries were pooled and then sequenced on an Illumina NovaSeq 6000 (Illumina) using a 150 bp paired‐end (PE) model, generating on average 30 million fragments and resulting in 60–70× mapped coverage with the Twist Exome v1.3 design. Sequencing reads were trimmed with FastP v.0.21.0 to remove low‐quality bases and adapter sequences and subsequently aligned to the human reference genome (GRCh38/hg38). Duplicates were removed and overlapping reads were clipped. Variants were identified with GATK Mutect2 v.4.2.4.1 (https://gatk.broadinstitute.org/hc/en‐us/articles/360037593851‐Mutect2) and filtered for sequencing artefacts and contamination using GATK FilterMutectCalls v4.2.4.1 (https://gatk.broadinstitute.org/hc/en‐us/articles/360036856831‐FilterMutectCalls). Functional annotation of gene variants was performed to describe their pathogenic effect, as reported in the Supplementary materials and methods.

### RNA sequencing

The RNA‐sequencing (RNA‐seq) data analysed in this study are publicly available in the Gene Expression Omnibus under accession number GSE287519 (https://www.ncbi.nlm.nih.gov/geo/query/acc.cgi?acc=GSE287519) and have been previously reported in detail [22].

In brief, strand‐specific RNA‐seq libraries were prepared from 400 ng of total RNA using the TruSeq RNA Sample Preparation Kit v2 (Illumina) following poly(A) capture. Libraries were size‐selected (500–750 bp) using a BluePippin system (Sage Science, Beverly, MA, USA) and sequenced on an Illumina NovaSeq 6000 platform (Illumina), generating approximately 33 million 150 bp PE reads per sample. Filtered reads were aligned to the *Homo sapiens* reference genome (Ensembl version 109, https://www.ensembl.org/info/website/archives/index.html; RRID:SCR_002344) using the STAR aligner (v2.7.9a) with the parameter – peOverlapNbasesMin 5. Expression counting and TPM (transcripts per million) calculation was performed as previously described [22]. Fusion transcripts of each cell line were identified from pooled RNA‐seq replicates (~100 million fragments each) using STAR‐Fusion v1.10.0 [23] and Arriba v2.1.0 [24] with default parameters. Only in‐frame fusion transcripts were considered.

AlphaFold3 (AF3) was used to predict the 3D structural features of the fusion proteins [25] and the DeepSite tool was employed to predict potential ligand‐binding sites within the chimeric protein [26], as detailed in the Supplementary materials and methods.

Detailed information on cell culture, human tumour biopsies, nucleic acid isolation, library preparation, sequencing, RT‐qPCR, FISH, MTS assay, western blotting, bioinformatics, statistical analysis, and *in silico* analysis is included in the Supplementary Materials and Methods. Details on primer sequences are reported in supplementary material, Tables S1 and S2.

## Results

### Comprehensive genomic profiling identifies clinically actionable variants in SN‐MM cell lines

By using the TSO500 NGS assay, we measured TMB, microsatellite instability (MSI), and genomic variants, including SNVs, insertions and deletions (InDels), and copy number variations (CNVs), in 523 cancer‐related genes. All SN‐MM cell lines were assessed as microsatellite stable (MSS), with a median of unstable microsatellite sites of 3.31% (range 1.69–5.22%; Figure 1A). The overall median TMB score per sample was 2.3 mut/Mb (range 1.6–4.7 mut/Mb; Figure 1A) and the median non‐synonymous TMB score was 1.57 mut/Mb (range 0.78–3.92 mut/Mb). Moreover, SN‐MM cell lines had a low genomic instability score (GIS; range 3–18; Figure 1A), indicating the absence of homologous recombination deficiency (HRD) status in these tumours. The TSO500 assay revealed a total of 50 variants, including SNVs and InDels, with a range of 6–14 variants per sample (Table 1 and supplementary material, Table S4). Of these, 42 variants were missense mutations, three frameshift insertions, three splice site mutations, and one was a nonsense mutation (Table 1 and supplementary material, Table S4). Among them, eight variants were defined as Tier I or Tier II, based on the VarSome Tiered classification system for somatic variants (Figure 1A and Table 1). Notably, *BRAF* p.Asn581Ser in SN‐MM1 and *NRAS* p.Gly12Asp in SN‐MM3 are potential clinically actionable variants in cancer treatment. Moreover, we found pathogenic mutations in tumour suppressor genes, including *PTEN* c.84T>G p.Ile28Met, *PTEN* c.106G>C p.Gly36Arg, and *TP53* c.374C>G p.Thr125Arg missense mutations in SN‐MM3; and *NF1* p.Glu1582ArgfsTer40 and *FANCA* p.Arg1187GlufsTer28 truncating mutations in SN‐MM1 and SN‐MM2 cell lines, respectively (Figure 1A and Table 1). A splice site variant of *TSC1* was also identified in SN‐MM1 and classified as Tier II (Figure 1A and Table 1). We also reported 12 somatic variants classified as Tier III and 30 somatic variants classified as Tier IV among SN‐MM cell lines (Figure 1A and supplementary material, Table S4). No pathogenic/likely pathogenic SNVs or InDels were identified in SN‐MM4 and SN‐MM5 cell lines.

**Figure 1:**
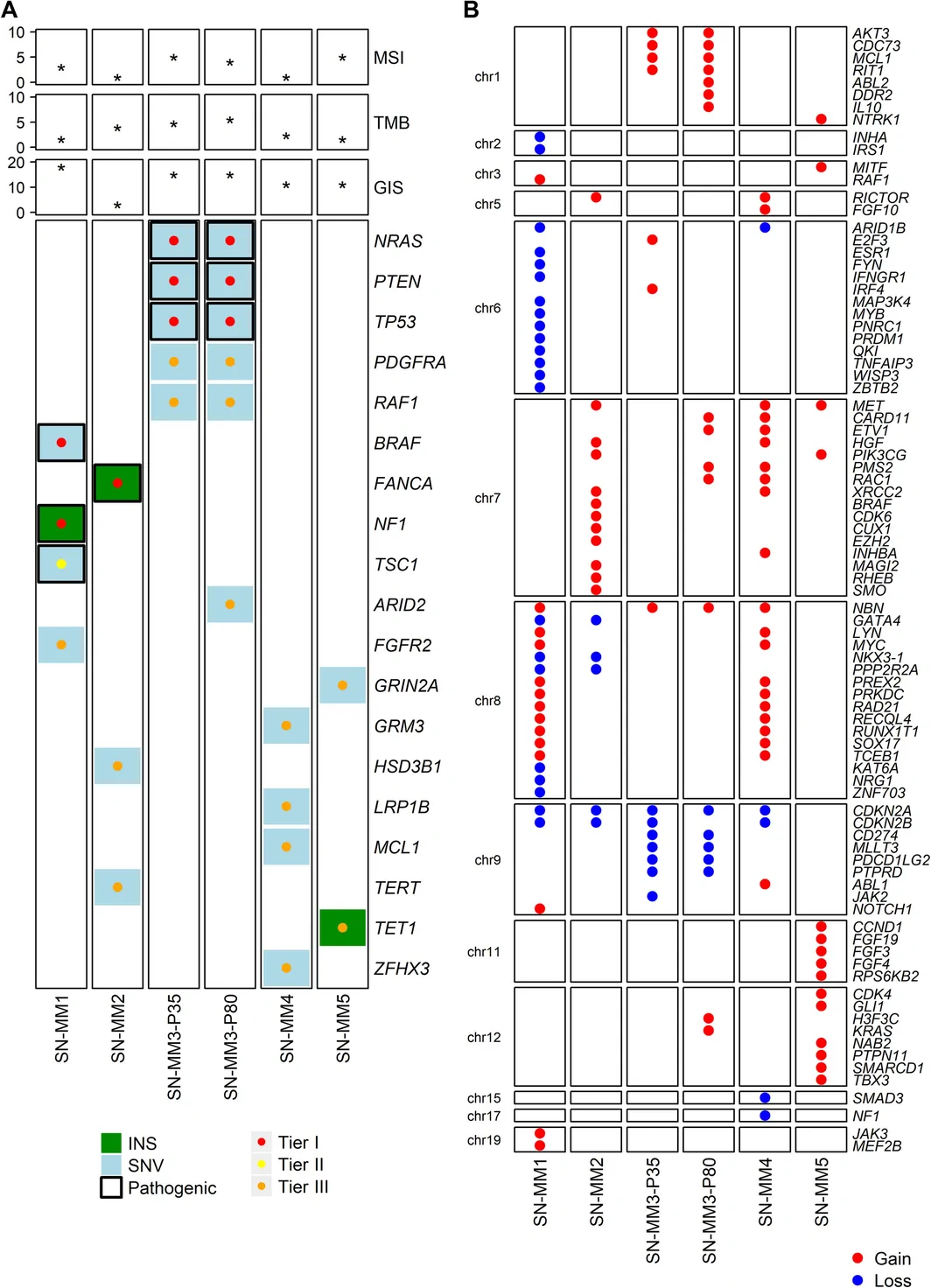
Oncoplots including the most frequently mutated genes in SN‐MM cell lines. (A) Oncoplot showing the MSI (percentage of unstable microsatellite sites), TMB (number of mutations/Mb), GIS (genomic instability score), SNVs (light blue), and InDels (green) in six cell line samples. Two different cell culture passages of the SN‐MM3 cell line (P35 and P80) are shown. Variants are classified as pathogenic/likely pathogenic (Tier I–II), based on the VarSome tiered classification system for somatic variants. VUS (Tier III) are also reported. (B) Plot showing gene CNVs among six cell line samples. Gene fold changes greater than 2 or less than 0.5 are reported as gains and losses, respectively.

**Table 1:** Pathogenic SNVs and InDels identified among SN‐MM cell lines by using target NGS panel.

Cell line sample	Chr	Start position	Gene	Exon	HGVSc	HGVSp	Variant classification	VAF (%)	COSMIC	ClinVar significance	VarSome
SN‐MM1	chr7	140453193	*BRAF*	15	c.1742A>G	p.Asn581Ser	Missense mutation	25	COSM462	Likely pathogenic	Tier I
SN‐MM1	chr9	135779797	*TSC1*	17	c.2041+1G>C	NA	Splice site	100	.	NA	Tier II
SN‐MM1	chr17	29592261	*NF1*	36	c.4743dup	p.Glu1582ArgfsTer40	Frameshift ins	45	NA	Pathogenic	Tier I
SN‐MM2	chr16	89811435	*FANCA*	36	c.3558dup	p.Arg1187GlufsTer28	Frameshift ins	64	NA	Pathogenic	Tier I
SN‐MM3	chr1	115258747	*NRAS*	2	c.35G>A	p.Gly12Asp	Missense mutation	66	COSM564	Pathogenic/likely pathogenic	Tier I
SN‐MM3	chr10	89653786	*PTEN*	2	c.84T>G	p.Ile28Met	Missense mutation	100	COSM87307	VUS	Tier I
SN‐MM3	chr10	89653808	*PTEN*	2	c.106G>C	p.Gly36Arg	Missense mutation	100	NA	Likely pathogenic	Tier I
SN‐MM3	chr17	7579313	*TP53*	4	c.374C>G	p.Thr125Arg	Missense mutation	100	COSM45243	Pathogenic/likely pathogenic	Tier I

ins, insertion.

Gains (fold change > 2) and losses (fold change < 0.5) of gene copy number were seen in 57 and 29 different cancer genes, respectively (Figure 1B). The most common amplifications were of *NBN* and *MET*, seen in three out of five SN‐MM cell lines. The highest fold change was measured for *MET* in SN‐MM5 at 18.8 (supplementary material, Table S5). Other genes showing copy number amplifications were *CCND1*, *FGF4*, *FGF19*, *FGF3*, and *CDK4* in SN‐MM5, with a fold change at 10.7, 10.2, 9.7, 8.3, and 6.5, respectively. Moreover, deep losses (fold change < 0.3) were observed for *CDKN2A* (fold change: 0.05 SN‐MM1; 0.04 SN‐MM2; 0 SN‐MM4; supplementary material, Table S5), as previously demonstrated [27], and *CDKN2B* (fold change: 0 SN‐MM1; 0.05 SN‐MM4; supplementary material, Table S5). These data suggest that SN‐MMs are characterized by frequent genomic rearrangements targeting a wide number of chromosome regions, as previously described [27], with a common consequence being the gain of oncogenes or the loss of tumour suppressor genes. The overall genomic landscape of SN‐MM indicates a low mutational burden but frequent large‐scale genomic rearrangements. It should be noted that two different sub‐culturing passages of the SN‐MM3 cell line (P35 and P80) display almost identical SNVs and InDels (Figure 1A), as well as a comparable CNV profile (Figure 1B), as proof of concept that the genomic profile remained stable over *in vitro* passages.

### WES of MM cells uncovers novel relevant SNVs in cancer‐associated genes

To further expand the analysis of the genomic landscape of SN‐MM cell lines, we applied a WES approach. A total of 24 somatic SNVs and six InDels, resulting in a VAF greater than 10%, were identified among SN‐MM cell lines, including the missense and truncating pathogenic mutations previously identified by the TSO500 assay (Table 2). We also found 11 novel somatic variants affecting cancer‐associated genes and predicted as of uncertain significance, classified as Tier III based on the VarSome classification system. Specifically, this set included the missense mutations of *ABLIM3* in SN‐MM1, *RYR1* in SN‐MM3, and truncating mutations of *MSH6* and *TYRP1* in SN‐MM3 (supplementary material, Table S6). The frameshift deletion in the melanocyte gene *TYRP1* has been previously identified in mucosal and acral melanomas [16, 27]; however, the mechanism by which this alteration contributes to melanoma pathogenesis remains unclear. All SNVs identified in the SN‐MM5 cell line were classified as Tier IV, based on the VarSome classification system. A large set of SNVs was validated by using Sanger sequencing on both cell lines and the corresponding parental tumour biopsies (Table 2 and supplementary material, Figure S1 and Tables S1 and S6). Moreover, *PTEN* c.84T>G was detected in both the SN‐MM3 cell line and tumour tissue by Sanger sequencing (supplementary material, Figure S1). Overall, these results further support the notion that this cellular system recapitulates parental tumours.

**Table 2:** List of pathogenic SNVs and InDels identified in SN‐MM cell lines by using WES approach.

Cell line sample	Chr position	Gene	Exon	HGVSc	HGVSp	Variant classification	VAF (%)	COSMIC	ClinVar	VarSome	Parental tumour^†^
SN‐MM1	7:140753393	*BRAF*	15	c.1742A>G	p.Asn581Ser	Missense mutation	22	COSM462	Likely pathogenic	Tier I	Yes
SN‐MM1	17:31265243	*NF1*	35	c.4680dupA	p.Glu1561Argfs*40	Frameshift ins	43		Pathogenic	Tier I	NA
SN‐MM2	16:89745027	*FANCA*	36	c.3558dupG	p.Arg1187Glufs*28	Frameshift ins	60		Pathogenic	Tier I	NA
SN‐MM3	1:114716126	*NRAS*	2	c.35G>A	p.Gly12Asp	Missense mutation	70	COSM564	Likely pathogenic	Tier I	Yes
SN‐MM3	10:87894051	*PTEN*	2	c.106G>C	p.Gly36Arg	Missense mutation	100		Pathogenic	Tier I	Yes
SN‐MM3	17:7675995	*TP53*	4	c.374C>G	p.Thr125Arg	Missense mutation	100	COSM45243	Likely pathogenic	Tier I	Yes

^†^ Validation of SNVs by using Sanger sequencing on the corresponding parental tumour biopsies.

ins, insertion; NA, not assessed.

By RNA‐seq analysis [22], we integrated expression data for mutated genes identified in SN‐MM cell lines to provide insight into their functional role (supplementary material, Table S7 and Figure S2). The missense mutations occurring in genes not expressed in SN‐MM cell lines (TPM < 5 across all samples) were considered unlikely to be biologically relevant. On the contrary, nonsense or frameshift mutations are expected to reduce transcript levels due to nonsense‐mediated decay [28]. Finally, genes showing significantly higher or lower TPM in the mutated SN‐MM cell line compared with wild‐type SN‐MM cell lines may provide preliminary indications to infer the functional impact of the variants of uncertain significance (Tier III/IV; supplementary material, Figure S2), although missense activating mutations may alter protein activity without affecting transcript abundance. Therefore, these findings require further investigation to understand their biological significance.

### Identification of novel oncogenic fusion transcripts in MM cells

Expressed in‐frame gene fusions involving at least one oncogene are described in various cancers and are shown to be the driver of malignant phenotypes. By RNA‐seq analysis, we found seven in‐frame fusions of gene transcripts among five SN‐MM cell lines (Table 3). These included four fusions involving different oncogenes: *CBFB*::*ALG8* in SN‐MM1, *MKRN1*::*BRAF* in SN‐MM2, *CCNB1*::*SCAMP1* in SN‐MM3, and *TRAF1*::*SUSD1* in SN‐MM4 (Table 3 and Figures 2 and 3). The expression of six out of seven fusion transcripts was confirmed by RT‐qPCR, which was performed on the corresponding SN‐MM cell lines at different *in vitro* culture passages (supplementary material, Figure S3A). For each fusion event, SN‐MM cell lines in which the corresponding fusion transcript was not identified by RNA‐seq were used as negative controls, showing no amplification (Ct values > 40). The *MKRN1*::*BRAF* fusion transcript is composed of exons 1–4 of *MKRN1* and exons 11–19 of *BRAF*, respectively (Figure 2A). Interestingly, the MKRN1::BRAF fusion protein lacks the N‐terminal regulatory region of BRAF, containing a RAS‐binding domain (RBD) and a cysteine‐rich domain (CRD), which maintains BRAF in an auto‐inhibited state, while retaining the Ser/Thr kinase domain of BRAF, likely leading to constitutive kinase activation (Figure 2A). The MKRN1 protein is truncated in the middle of the MKRN‐type zinc finger CH domain, resulting in the loss of the C‐terminal C3H zinc finger domain and ring finger domain with E3 ubiquitin ligase activity, whereas three C3H zinc finger domains and a PAM2‐like domain with RNA‐binding activity are retained (Figure 2A,B). Importantly, *in silico* analyses indicated that fusion with MKRN1 does not interfere with MEK dimerization (Figure 2C), a key step in MAPK cascade signalling. By using a dual‐colour, break‐apart FISH assay combined with chromosome 7 centromeric probes, we detected evidence of *BRAF* gene rearrangements in conjunction with chromosome 7 polysomy in the majority of tumour cells from the SN‐MM2 cell line and the corresponding parental tumour (supplementary material, Figure S4), supporting our finding of *MKRN1*::*BRAF* fusion. Consistently, we analysed a retrospective SN‐MM cohort (*n* = 10) [22] using the break‐apart FISH assay and revealed *BRAF* rearrangements in 50% of cases; however, in three of these cases, the rearrangement was present only in a subclonal fraction of tumour cells (Table 4 and supplementary material, Figure S4). These findings support the notion that *BRAF* structural alterations are recurrent events in SN‐MM and reinforce the biological relevance of MAPK‐directed therapeutic strategies.

**Table 3:** List of fusion transcripts identified in SN‐MM cell lines by using RNA‐seq approach.

Cell line	Fusion transcript	Left breakpoint	Right breakpoint	Fusion annotation
SN‐MM1	*CBFB*::*ALG8*	16:67036755	11:78101195	Gene 1: Oncogene
SN‐MM2	*MKRN1*::*BRAF*	7:140459007	7:140781693	TCGA Fusion
SN‐MM3	*CCNB1*::*SCAMP1*	5:69175537	5:78388837	Gene 1: Oncogene
SN‐MM3	*CRLS1*::*MCM8*	20:6015490	20:5954608	/
SN‐MM3	*LDHB*::*C2CD5*	12:21641952	12:22457161 12:22459542	/
SN‐MM4	*TRAF1*::*SUSD1*	9:120909230	9:112041960	Gene 1: Oncogene
SN‐MM5	*MBNL1*::*ITGA2*	3:152300367	5:53048363	/

**Figure 2:**
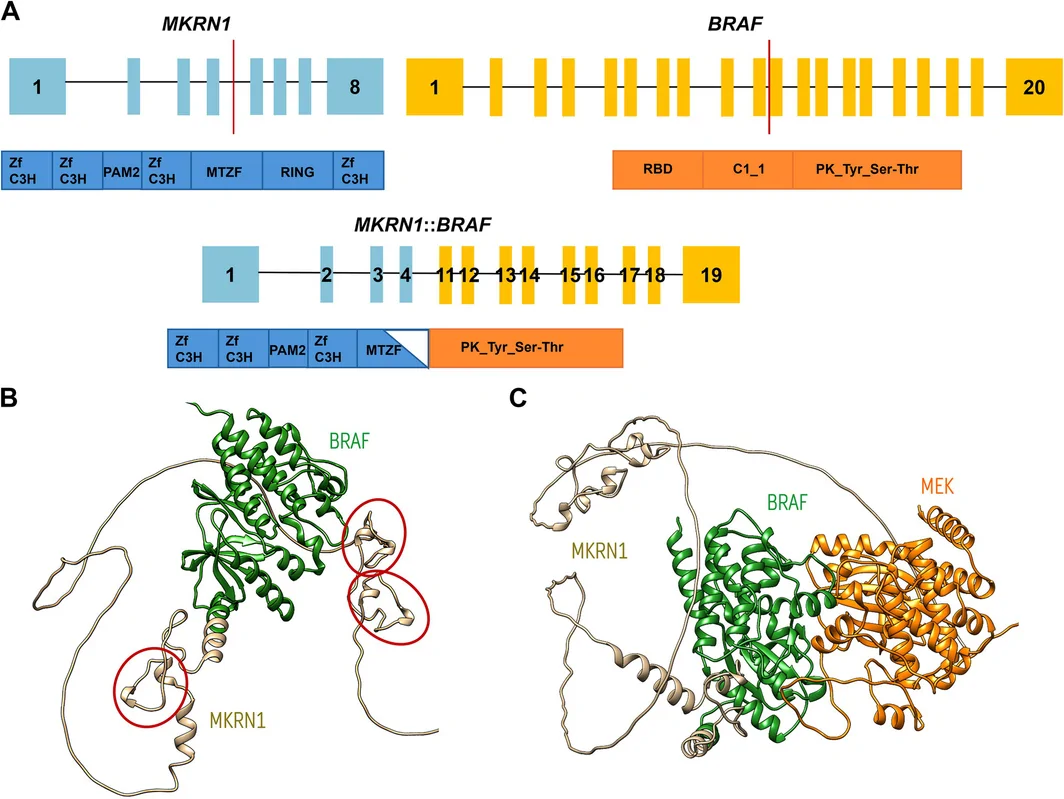
*MKRN1*::*BRAF* fusion transcript and structural prediction of the corresponding fusion protein. (A) *MKRN1* and *BRAF* wild‐type genes and proteins (upper panel), and the fusion transcript and corresponding chimeric protein (lower panel) are shown. Exons of *MKRN1* and *BRAF* are represented by light blue and orange blocks, respectively. Gene breakpoints are indicated by vertical red lines. Protein domains of MKRN1 and BRAF are depicted as blue and orange‐red rectangles, respectively. Zf C3H: CCCH‐type zinc finger; MTZF: MKRN‐type zinc finger; RING: C3HC4‐type zinc finger; RBD: Raf‐like RAS‐binding domain; C1_1: phorbol ester/diacylglycerol binding domain; PK_Tyr_Ser‐Thr: protein tyrosine and serine/threonine kinase. (B, C) *In silico* structural prediction generated with AF3. C3H‐type zinc finger of the MKRN1::BRAF fusion protein are highlighted with red circles in panel B. The complex of the MKRN1::BRAF fusion protein with MEK is shown in panel C. The RMSD between AF3 prediction of BRAF::MEK heterodimer and PDB ID 7M0Y is 0.545 Å.

**Figure 3:**
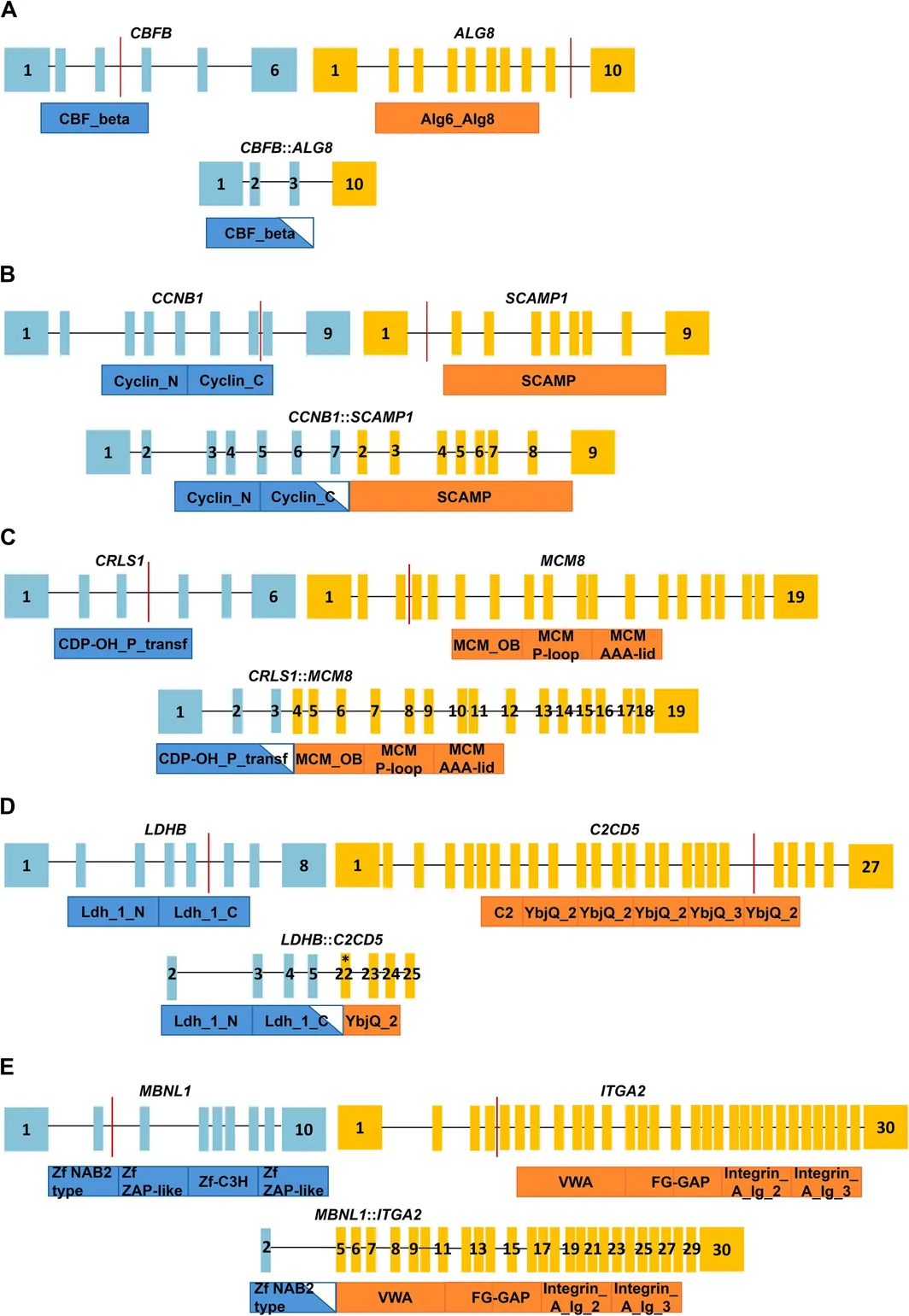
Fusion transcripts identified among SN‐MM cell lines. Wild‐type genes and proteins are shown in the upper part of each panel. The fusion transcripts and chimeric proteins are shown in the lower part of each panel. Exons of the ‘gene A’ and ‘gene B’ involved in the fusion transcripts are represented by light blue and orange blocks, respectively. Gene breakpoints are indicated by vertical red lines. Protein domains are depicted as blue and orange‐red rectangles, respectively. (A) *CBFB*::*ALG8*. CBF_beta: core binding factor beta subunit; Alg6_Alg8: ALG6, ALG8 glycosyltransferase family. (B) *CCNB1*::*SCAMP1*. Cyclin_N: cyclin N‐terminal domain; Cyclin_C: cyclin C‐terminal domain; SCAMP: secretory carrier‐associated membrane protein family. (C) *CRSL1*::*MCM8*. CDP‐OH_P_transf: CDP‐alcohol phosphatidyltransferase; MCM OB domain; MCM P‐loop domain; MCM AAA‐lid domain. (D) *LDHB*::*C2CD5*. Ldh_1_N: lactate/malate dehydrogenase, NAD binding domain; Ldh_1_C: lactate/malate dehydrogenase, alpha/beta C‐terminal domain; C2 domain; YbjQ_2: C2 domain‐containing protein 5, YbjQ‐like domain; YbjQ_3: C2 domain‐containing protein 5, fifth YbjQ‐like domain. (E) *MBNL1*::*ITGA2*. Zf_NAB2‐type: RNA‐binding, Nab2‐type zinc finger; Zf_ZAP‐like: ZAP‐like CCCH zinc finger; Zf‐C3H: zinc finger C‐x8‐C‐x5‐C‐x3‐H type; VWA: von Willebrand factor type A domain; FG‐GAP repeat; Integrin_A_Ig_2: integrin alpha Ig‐like domain 2; Integrin_A_Ig_3: integrin alpha Ig‐like domain 3.

**Table 4:** FISH detection of *BRAF* rearrangement in a restrospective MM cohort (*n* = 10).

Patient	Age (years)	Gender	Subsite	FISH % of positivity	FISH signal pattern
#1	75	Female	Nasal cavity	0%	F + single 3′ *BRAF*
#2	76	Female	Paranasal sinuses	0%	F + single 3′ *BRAF*
#3	74	Male	Paranasal sinuses	15%	F + single 3′ *BRAF*
#4	86	Female	Nasal cavity	20%	F + single 3′ *BRAF*
#5	71	Female	Nasopharynx	0%	F + single 3′ *BRAF*
#6	84	Female	Nasal cavity	0%	F + single 3′ *BRAF*
#7	59	Male	Nasal cavity	85%	F + single 3′ *BRAF*
#8	75	Female	Nasal cavity	0%	F + single 3′ *BRAF*
#9	78	Male	Nasal cavity	15%	F + single 3′ *BRAF*
#10	74	Male	Nasal cavity	85%	F + single 3′ *BRAF*

F, fusion signals.

In the SN‐MM1 cell line, we identified an in‐frame fusion of the *CBFB* and *ALG8* genes (Figure 3A). The *CBFB* gene encodes the beta subunit of the core‐binding factor transcription factor complex and heterodimerizes with the alpha subunit, a RUNX protein, to increase its DNA‐binding affinity and regulate cell proliferation, differentiation, and apoptosis. The ALG8 protein is an alpha‐3‐glucosyltransferase required for protein *N*‐glycosylation, but its catalytic domains are lost in the fusion protein (Figure 3A). The predicted structure of the fusion protein truncates CBFB at residue 94, removing critical interface residues, including the hotspot residue Asn104, which is essential for binding RUNX1/CBFα (Figure 4A and supplementary material, Figure S3B) [29].

**Figure 4:**
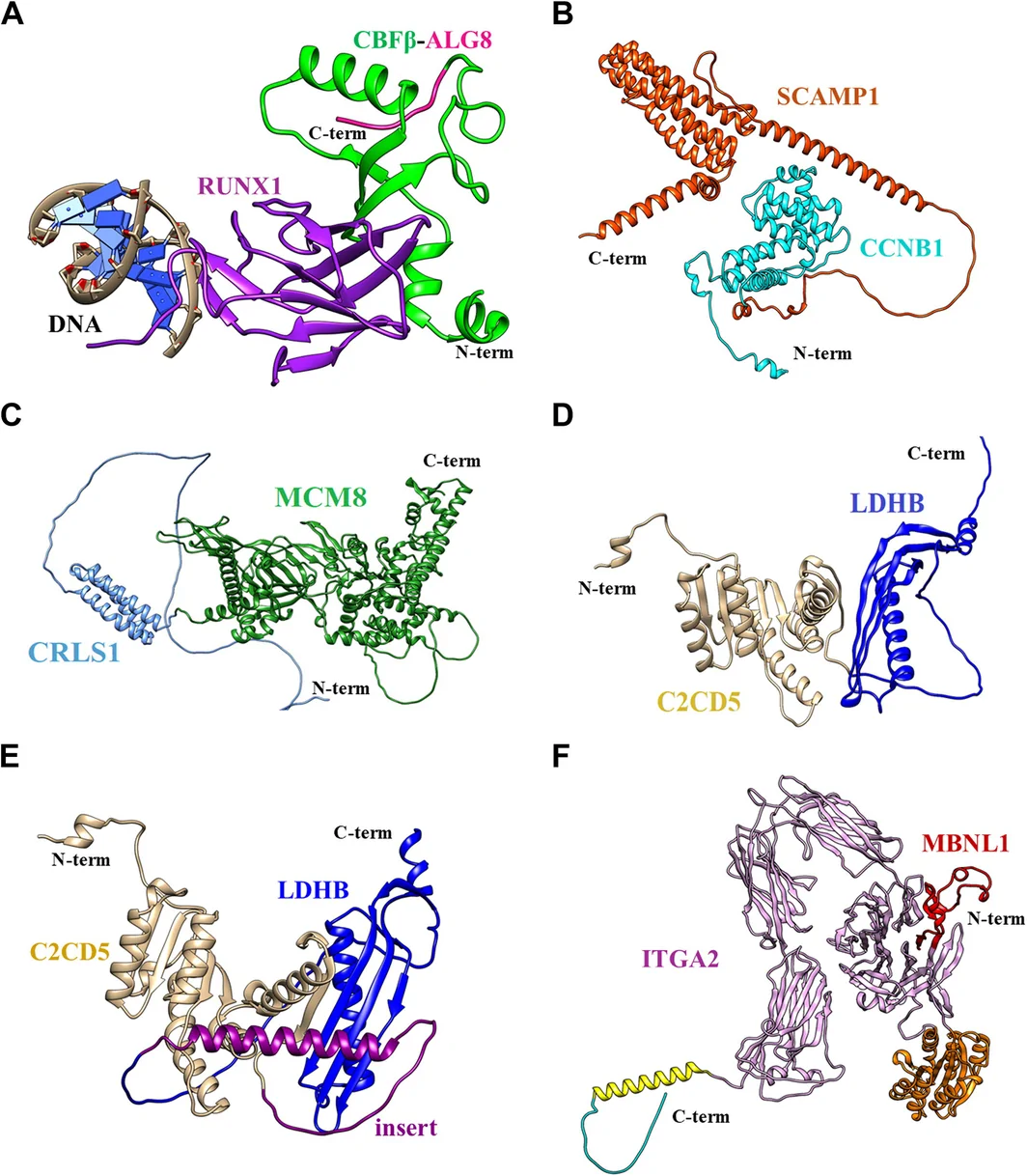
Structural predictions of fusion products identified among SN‐MM cell lines. *In silico* structural predictions were generated with AF3. (A) CBFB (green)::ALG8 (fuchsia) fusion protein in complex with RUNX1 (lilac) and DNA. (B) SCAMP1 (reddish‐brown)::CCNB1 (cyan) fusion protein. (C) CRLS1 (light blue)::MCM8 (forest green) fusion protein. (D) LDHB (blue)::C2CD5 (gold) first breakpoint, C2CD5 isoform 1. (E) LDHB (blue)::C2CD5 (gold) second breakpoint, C2CD5 isoform 3 with insert of 52 amino acids in purple. (F) MBNL1 (red)::ITAG2 fusion protein. Three main domains of ITGA2 protein are shown: extracellular domain (residues 30–1,132; pink), transmembrane domain (residues 1,133–1,154; yellow), and a cytoplasmic domain (residues 1155–1181; cyan). In addition, the α2 chain contains the interactive/inserted domain (I domain; orange), located within the extracellular region and beginning at residue 140.

We also identified *CCNB1*::*SCAMP1*, *CRSL1*::*MCM8*, and *LDHB*::*C2CD5* fusions in the SN‐MM3 cell line. The *CCNB1*::*SCAMP1* fusion retained the cyclin N‐terminal domain of CCNB1, whereas the C‐terminal domain was truncated; additionally, two of the three NPDxD motifs of the secretory carrier‐associated membrane protein SCAMP1 [30] were lost (Figures 3B and 4B). A distinct fusion event involved the mitochondrial cardiolipin synthase 1 (*CRLS1*) and the helicase *MCM8*, which has been recently detected in the mitochondrial matrix [31] (Figures 3C and 4C). MCM8 interacts with its partner MCM9 to form a ring‐shaped complex involved in DNA replication. The fusion protein retains the CRSL1 catalytic domain but is truncated at amino acid 191, losing three out of six predicted transmembrane α‐helices, while MCM8 lacks the first N‐terminal 84 amino acids, which are not involved in the interaction with MCM9, as supported by structural superposition between the AF3‐predicted model and the MCM8–MCM9 ring complex (supplementary material, Figure S3C). Another fusion transcript involved the *LDHB* gene, a subunit of lactate dehydrogenase (LDH) that functions as a heterotetramer with its partner LDHA, and the *C2CD5* gene (Figure 3D). The predicted LDHB::CDCD5 fusion protein was depleted of the α‐helix that seals the NADH binding site and the entire substrate‐binding domain, likely compromising LDHB enzymatic activity (Figure 4D,E). Moreover, deletion of the first 844 residues of C2CD5 resulted in the loss of both C2 Ca^2+^‐binding domains and all the known phosphorylation sites (Figure 4D,E). Notably, two distinct LDHB::C2CD5 isoforms were identified, sharing the same LDHB sequence but incorporating either C2CD5 isoform 1 or 3 (Figure 4D,E).

In SN‐MM5, we identified an MBNL1::ITGA2 fusion (Figure 3E). MBNL1, an alternative splicing regulator with an important role in muscle development and function [32], preserved only one C3H‐type zinc finger domain in the fusion. The *ITGA2* that encodes the integrin α2 subunit, which forms a primary receptor for collagen (α2β1), lacks the first 129 residues but retains the extracellular I domain known to be involved in collagen binding [33] (Figures 3E and 4F). Although the extracellular I domain is structurally preserved, the functional implications on collagen recognition remain uncertain.

Although the prevalence of these fusion transcripts in larger SN‐MM cohorts remains to be established, their predicted structural consequences, such as the loss of regulatory domains and retention of kinase or cell cycle‐related domains, suggest potential oncogenic activity.

### Relevance of recurrent and constitutive MAPK pathway activation in SN‐MM cell lines

SN‐MM cell lines are characterized by multiple genomic alterations, including recurrent MAPK‐activating alterations (e.g. *BRAF* p.N581S, *NRAS* p.G12D, *NF1* truncation, and *MKRN1*::*BRAF* fusion). Therefore, we hypothesized that SN‐MM cells may rely on MAPK signalling for proliferation and survival. Based on these premises, we explored MAPK signalling activation in SN‐MM cell lines, measuring ERK1/2 protein expression and phosphorylation by western blotting. Constitutive activation of pERK1/2 protein was detected in all SN‐MM cell lines (Figure 5A).

**Figure 5:**
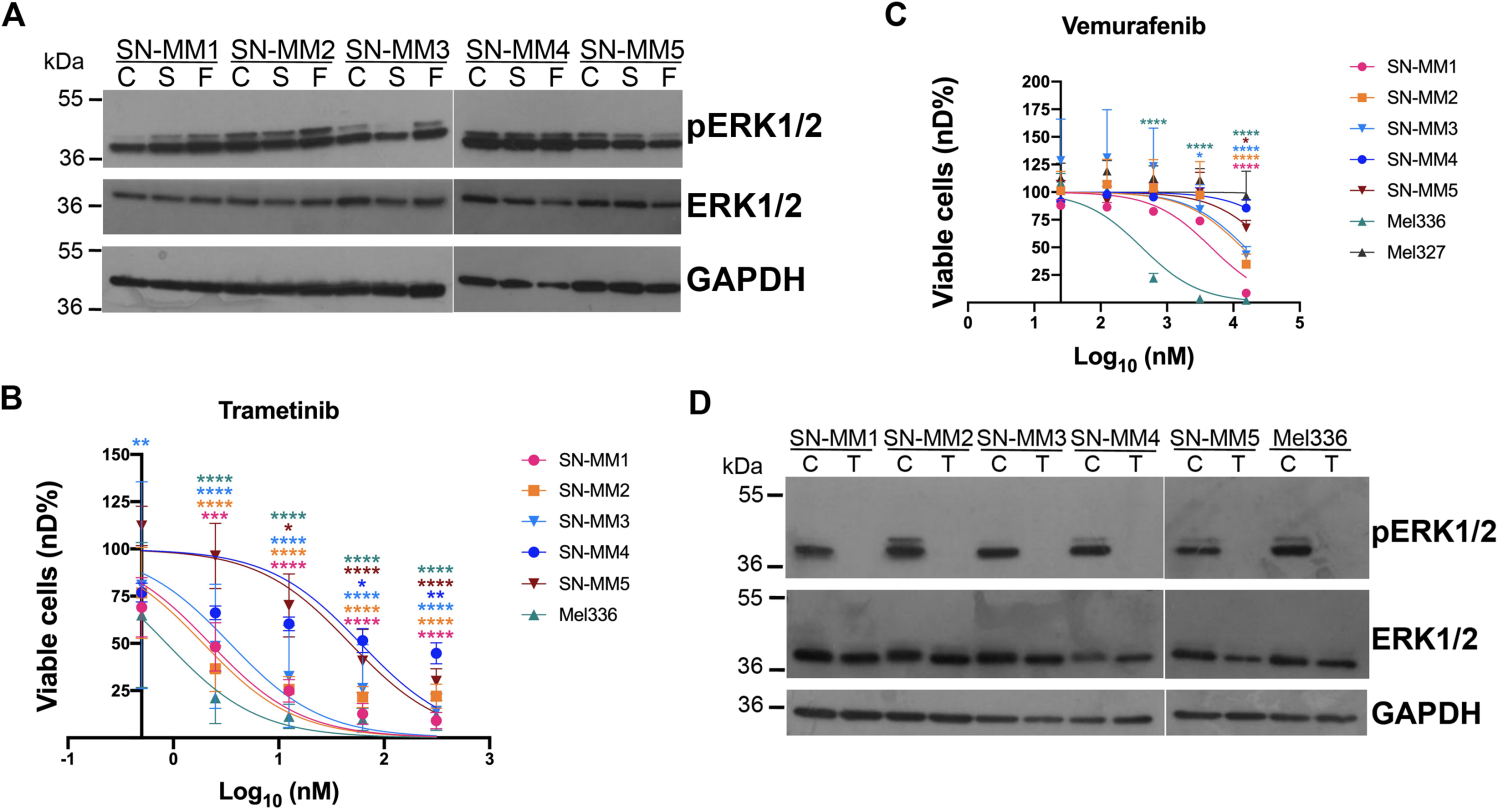
MAPK pathway activation in SN‐MM cell lines and treatment with BRAFi and MEKi. (A) Western blotting showing ERK1/2 and pERK1/2 (Thr202/Tyr204) expression in SN‐MM cell lines cultured in complete RPMI medium (C), after overnight starvation with 1% FBS (S), and replenished with 10% FBS for 30 min (F) before cell lysis. GAPDH was used as a housekeeping control. (B, C) Dose–response curves of trametinib and vemurafenib in SN‐MM cell lines. SN‐MM cells were treated with (B) trametinib (0.5–312.5 nm) for 3 days (SN‐MM2; SN‐MM3; SN‐MM5) or 6 days (SN‐MM1; SN‐MM4) or with (C) vemurafenib (25–15.625 μm) for 6 days. Results are expressed as percentages of viable cells versus untreated cells ± SD of four independent experiments, run in triplicate. Two‐way ANOVA statistical analysis and Bonferroni's multiple comparison *post hoc* test were performed. For treated cells versus untreated cells; *p < 0.05; **p < 0.01; ***p < 0.001; ****p < 0.0001. (D) Western blotting showing ERK1/2 and pERK1/2 expression in SN‐MM cell lines cultured in complete RPMI medium (C) ± trametinib (50 nm; t) for 1 h. GAPDH was used as the housekeeping control.

To address the role of the MAPK pathway in SN‐MM cell proliferation and survival, cell lines were treated with increasing concentrations of the BRAF inhibitor (BRAFi) vemurafenib (25–15.6 μm) or the MEK inhibitor (MEKi) trametinib (0.5–312.5 nm) for 6 days. The mel‐336 cutaneous melanoma cell line bearing *BRAF* p.Val600Lys was used as a positive control. The dose–response curves revealed that trametinib induced a decreased viability in all SN‐MM cell lines, although with different sensitivity (Figure 4B). Sigmoidal concentration–response function was applied to calculate the IC_50_ values of trametinib, ranging from 0.9 to 59.6 nm among different cell lines (supplementary material, Table S8). The mel‐336 cell line displayed an IC_50_ within this expected range [34, 35]. Trametinib was highly active in inhibiting cell proliferation and survival in three out of five SN‐MMs, as its efficacy reached about 80–90% at the highest concentration tested (supplementary material, Table S8). On the contrary, vemurafenib treatment significantly affected the viability of three out of five SN‐MMs only at the highest dose (15.6 μm), compared with mel‐336 (0.6 μm) (Figure 4C and supplementary material, Table S8). Vemurafenib reached maximum efficacy in SN‐MM1 and SN‐MM2 that was about 91% and 65%, the highest concentration tested (supplementary material, Table S8). Since vemurafenib is known to be ineffective in the absence of *BRAF* V600 mutations, we used the mel‐327 cutaneous melanoma cell line, which is *BRAF* wild‐type, as a negative control. These findings confirm that MAPK activation in MM is partially sustained by *BRAF* molecular alterations, such as *BRAF* p.N581S and *MKRN1*::*BRAF* fusion that increased the Ser/Thr kinase activity, and by oncogenic co‐alterations such as *NRAS* p.G12D and *NF1* truncation that activate MAPK upstream of BRAF.

The interaction motif of the small molecule inhibitor dabrafenib with BRAF was investigated by means of molecular docking. To achieve this, a docking simulation which encompasses a conformational search and an energy scoring step was carried out. Molecular docking of dabrafenib on BRAF showed that the Asp581Ser mutant induces a change in the conformation of the ligand pose and a decrease in the calculated binding energy, from −10.6 kcal/mol for wild‐type BRAF to −8.4 kcal/mol (supplementary material, Figure S5A,B). In the current study, the simulations suggest that dabrafenib should bind the Asp581Ser mutant less efficiently with respect to wild‐type BRAF. In contrast, for the p.Val600Glu mutant, the docking results were only marginally affected by the mutation compared with the wild‐type protein, as the mutated residue is located outside the defined docking grid (supplementary material, Figure S5C,D). A similar behaviour was observed with the inhibitor vemurafenib: the molecular docking on BRAF showed a decrease in the calculated binding energy, from −11.8 kcal/mol for wild‐type BRAF to −9.2 kcal/mol for the p.Asp581Ser mutant, and a change in the conformation of the final pose that shows a departure from the mutated residue (supplementary material, Figure S5E,F). Thus, these data support a lower efficacy of BRAFi in tumour cells bearing the *BRAF* p.Asp581Ser (e.g. SN‐MM1) mutation versus the *BRAF* p.Val600Glu (e.g. mel‐336) mutation. Subsequently, DeepSite analysis was performed to model the MKRN1::BRAF structure. By comparing the predicted binding cavities with those identified in the wild‐type BRAF structure, the potential binding pocket resulted in a larger size and a different shape (supplementary material, Figure S5G–J). Molecular docking showed that dabrafenib and vemurafenib preferentially target an additional pocket in the fusion transcript at the interface between the two fusion peptides. This shift suggests that these BRAF inhibitors could act through a different mechanism with respect to the canonical direct BRAF inhibition. Finally, trametinib totally inhibited ERK1/2 phosphorylation in all SN‐MM cell lines after 1 h of treatment (Figure 4D). These findings, together with the genomic profile of SN‐MM cell lines, are in keeping with the relevant role of the activated MAPK pathway in MM.

## Discussion

The rarity of MMs together with the lack of preclinical models has limited our understanding of their genomic landscape and corresponding drug targets. The present study expands on the genomic landscape of MM by analysing patient‐derived cell lines [21] using genomic‐scale sequencing approaches.

The 8th edition of the *AJCC Cancer Staging Manual* has identified MM as a distinct clinicopathological entity, and its clinical and molecular profiles are largely different from those of CM, with most MMs falling into the triple‐WT subtype [8, 16]. Recent sequencing studies showed that MM has a lower burden of somatic mutations but accumulates complex structural genomic rearrangements and chromosomal instability compared with CM [9, 10, 14]. In particular, MMs have a high number of SVs, kataegis loci, and complex localized events, such as chromothripsis, frequently accompanied by focal amplifications and deletions targeting oncogenes and tumour suppressor genes [11, 15]. The overall genomic landscape of MM sustains the biological significance of these large‐scale genomic rearrangements in the majority of cases [15]. Overall, the genomic and functional features observed in our five patient‐derived SN‐MM cell lines closely resemble those reported in recent large‐scale sequencing studies of primary and metastatic SN‐MM [18, 19, 20], reinforcing the relevance of our model system and supporting the generalizability of our findings. Besides the identification of pathogenic variants of well‐documented driver genes in melanoma, such as *BRAF*, *NRAS*, *NF1*, *PTEN*, and *TP53*, our sequencing data also unveiled variants in genes encoding for protein known to activate the MAPK pathway (i.e. *RAF1*, *ERBB2*). In addition, WES analysis identified VUS mutations on known and emerging cancer genes, such as *ABLIM3*, *RYR1*, *MSH6*, and *TYRP1*, requiring functional validation. Correlations between gene expression and mutational status may provide preliminary indications, but they are not sufficient to infer the functional impact of the newly identified variants.

We have previously examined the CNV features of SN‐MM by using array‐based comparative genomic hybridization [21]. The TSO500 NGS approach also provided the opportunity to assess the oncogenic CNV landscape of SN‐MM. We identified recurrent gains of *MET* and *NBN*, and losses of *CDKN2A* [21] and *CDKN2B*. Moreover, SN‐MM2 harbours the *CDK6* amplification, while SN‐MM5 harbours *CDK4* and *CCND1* amplifications [21]. *CDK4* encodes the catalytic subunit of the cyclin‐dependent protein kinase complex and can form a complex with CDK6/cyclin D1, which targets RB1 and controls cell‐cycle G1/S phase progression. CDK4 is also the inhibitory binding partner of CDKN2A p16^INK4A^. Hence, their amplification may result in deregulation of the p16/cyclin D/CDK4/pRB pathway and might play an important role in MM development. Melanomas with wild‐type *BRAF* or *NRAS* frequently had increases in the number of copies of *CDK4* and *CCND1*, downstream components of the RAS–BRAF pathway [36]. Notably, a patient‐derived xenograft trial demonstrated robust antitumour effects of the CDK4/6 inhibitor palbociclib in MMs harbouring *CDK4* amplification [15].

The majority of SN‐MM cell lines display genomic aberrations in genes of the cell‐cycle metaprogram, MAPK, and PI3K pathways, leading to the cancer hallmarks of sustained proliferative signalling and evading growth suppression [14]. However, the mechanism by which these pathways are altered differs between samples. We also observed that the melanocyte development and pigmentation signalling pathway (including *MITF* gain, *TYRP1* frameshift deletion, and *SOX10* non‐synonymous mutation) is altered in SN‐MM, suggesting that alterations in genes involved in melanocyte survival and melanogenesis might contribute to melanoma progression [37].

Among the relevant findings that emerged from this study, the SV in SN‐MM cell lines included six different fusion events. This is consistent with the large‐scale genomic analysis reporting hundreds of rare fusion events in sinonasal and oral mucosal melanomas [11], highlighting the extensive genomic rearrangements that characterize these tumours. Among the fusion transcripts detected in our SN‐MM cell lines, *MKRN1*::*BRAF* fusions have been reported in different solid tumours as rare but recurrent oncogenic drivers [38], and *CBFB* has been previously found to be fused to the *MYH11* gene, a known oncogenic fusion in acute myeloid leukaemia [39]. Although extremely uncommon, *MKRN1*::*BRAF* fusion, and specifically the exon 4–exon 11 variant, has been reported in colorectal adenocarcinoma and head and neck neuroendocrine carcinoma [38]; in oesophageal gastrointestinal stromal tumour showing low or absent KIT expression [40]; in low‐grade serous ovarian cancer [41]; in pilocytic astrocytoma, where whole‐genome sequencing of 96 cases identified a single *MKRN1*::*BRAF* fusion [42]; in one anaplastic thyroid carcinoma cell line; and in papillary thyroid carcinoma with an estimated frequency of 0.2% [43]. Moreover, the partner genes involved in the six fusion products regulate various biological processes relevant to cancer fitness. CCNB1 promotes cell proliferation, migration, and invasion [44, 45]; MCM8 is implicated in genomic instability by regulating multiple DNA‐related processes, including DNA replication, homologous recombination, and mismatch repair [46]; and LDHB promotes cancer cell metabolism, growth, and metastatic dissemination [47].

Based on published studies on *BRAF* fusions and by using published models for confirming activation and prediction of the protein amino acid sequences, we expect the novel *MKRN1*::*BRAF* fusion to be similarly oncogenic. *BRAF* fusion genes represent an alternative mechanism of MAPK pathway activation and have been identified in a small percentage of triple wild‐type CM and across distinct tumour types [38, 40, 41, 42, 43]. All *MKRN1*::*BRAF* fusions described to date retain the intact BRAF kinase domain and lead to potent MEK/ERK activation. The *MKRN1*::*BRAF* fusion has also been reported as an acquired resistance mechanism in an EGFR‐mutant non‐small cell lung cancer patient treated with osimertinib, suggesting a useful biomarker to identify patients who may benefit from combined BRAFi + MEKi therapy as a strategy to overcome resistance [48]. MKRN1 is involved in regulation of energy metabolism, tumour suppression, the cell cycle, and apoptosis by degrading their protein substrates, such as FADD, hTERT, p53, p21, PPARγ, and AMPK [49, 50, 51]. Notably, the *BRAF* fusion with *MKRN1* does not interfere with its MEK dimerization but directly engages MEK and effectively transduces the signal, consistent with trametinib sensitivity [52, 53]. In this context, trametinib appears to target the BRAF/MEK complex rather than free active MEK. It stabilizes the MEK activation loop in a conformation that is resistant to BRAF‐mediated dual phosphorylation, which is necessary for full MEK activation [53]. Our observation that *MKRN1*::*BRAF* fusion in SN‐MM2 confers sensitivity to trametinib *in vitro* is consistent with clinical and preclinical evidence showing meaningful clinical activity of trametinib in metastatic melanomas harbouring *BRAF* fusions [54], although responses vary according to the specific fusion and its expression level [55]. Altogether, these findings strongly support the notion that the MKRN1::BRAF fusion product is a therapeutic targetable oncogenic driver, although therapeutic responses are heterogeneous and may require tailored MAPK‐directed strategies [54, 55]. As an additional biologically relevant SV, the LDHB–C2CD5 fusion proteins preserve the C‐terminal portion of C2CD5 previously shown to bind CDK5 and FIBP proteins implicated in cell proliferation and migration, suggesting that this complex plays a role in promoting tumourigenic behaviour [56].

The heterogeneous response to BRAFi/MEKi observed across the five SN‐MM cell lines likely reflects their underlying genomic diversity, consistent with multiple mechanisms of MAPK pathway activation in cancer; therefore, only a molecularly defined subset of SN‐MMs may benefit from MAPK‐directed therapies. Clinical stratification should consider comprehensive molecular profiling to classify *BRAF* alterations, *NRAS* and *NF1* status, and relevant co‐alterations (i.e. PI3K/PTEN or CDK4/CCND1). While *BRAF* V600 tumours are candidates for BRAFi ± MEKi, non‐V600 *BRAF* variants and *BRAF* fusions require functional validation before MAPK‐targeted therapy can be recommended [57, 58]. From a diagnostic perspective, the FISH assay can be readily implemented in routine pathology to identify *BRAF* fusions in MM patients. In FISH‐positive cases, the specific *BRAF* fusion partners could be resolved using targeted next‐generation sequencing, while targeted RNA sequencing may provide molecular and functional validation, particularly in cases where the biological impact of the rearrangement is uncertain. *NRAS*‐mutant or *NF1*‐loss tumours may be considered for MEK‐directed strategies, ideally within clinical trials or combination approaches [59]. Our findings provide proof of concept but are insufficient for definitive clinical stratification, and prospective validation in larger cohorts and additional functional studies are required.

In conclusion, we have identified a range of new and significant MM variants that affect multiple signalling pathways relevant for cancer progression. This suggests that mucosal melanocytes take diverse paths as they transform and progress. Nevertheless, our findings require further genetic and chemical validation to confirm their biological function and identify potential biomarkers. The mutational profiles of most MMs suggest that they may be susceptible to several targeted therapies, such as PI3K, CDK4/6, or MEK inhibitors [11]. Finally, our study also supports the use of MAPK blockade as another promising treatment strategy.

## Author contributions statement

WV conceived and designed the study, supervised data analysis, supervised the study. MM conceived and designed the study, collected and analyzed the data. MBR, collected and analyzed the data, performed bioinformatics, *in silico*, and statistical analysis, interpreted the results and wrote the manuscript. MO collected and analyzed the data, performed bioinformatics, *in silico*, and statistical analysis. LB collected and analyzed the data, performed bioinformatics, *in silico*, and statistical analysis. SP collected and analyzed the data, carried out *in vitro* experiments. IP collected and analyzed the data, carried out *in vitro* experiments. GR collected and analyzed the data, performed bioinformatics, *in silico*, and statistical analysis. AB collected and analyzed the data. EC collected and analyzed the data. PB collected and analyzed the data. VP carried out *in vitro* experiments. ADM carried out *in vitro* experiments. DG performed bioinformatics, *in silico*, and statistical analysis. LA provided facility and acquired the data, supervised data analysis. DM provided facility and acquired the data, supervised data analysis. GP provided facility and acquired the data. MR provided facility and acquired the data. GEM supervised data analysis, interpreted the results and wrote the manuscript. AG supervised data analysis. MR supervised data analysis. DL acquired and managed patients. GEM interpreted the results and wrote the manuscript. All authors read and approved the final manuscript.

## Supporting information


Supplementary materials and methods
**Figure S1.** Validation of SNVs in SN‐MM cell lines and corresponding parental tumours by Sanger sequencing
**Figure S2.** Expression levels of mutated genes across SN‐MM cell lines
**Figure S3.** Expression of fusion transcripts and structural modelling of selected fusion proteins in SN‐MM
**Figure S4.** Interphase FISH detection of *BRAF* rearrangement
**Figure S5.** Molecular docking analysis of dabrafenib and BRAF variants
**Figure S6.** Full western blotting images corresponding to those shown in Figure 5
**Table S1.** List of PCR primers for genes evaluated by Sanger sequencing on SN‐MM cell lines and corresponding parental tumours
**Table S2.** List of PCR primers for fusion transcripts evaluated by RT‐qPCR
**Table S3.** Values of pTM score obtained for the studied models (referred to in Supplementary materials and methods)
**Table S4.** SNVs and InDels of unknown clinical significance identified in SN‐MM cell lines by using target NGS panel
**Table S5.** Fold change of the gene copy number variations in SN‐MM cell lines
**Table S6.** List of novel SNVs and InDels identified among the SN‐MM cell lines by WES
**Table S7.** Gene expression level (TPM) by RNA‐seq analysis across SN‐MM cell lines
**Table S8.** Efficacy of MEKi and BRAFi treatment in SN‐MM cell lines

## Data Availability

All data relevant to the study are included in this published article or have been uploaded as supplementary information files. The WES data generated and analysed in this study have been submitted to NCBI (https://www.ncbi.nlm.nih.gov/sra/?term=PRJNA1356825). The RNA‐seq data generated and analysed in this study are publicly available in the Gene Expression Omnibus (GEO) (https://www.ncbi.nlm.nih.gov/geo/) under accession number GSE287519.
