# Supplementary material for: Comprehensive genomic profiling of mucosal melanoma reveals novel fusion transcripts and dysregulation of cell‐cycle, MAPK, and PI3K pathways
Source: J Pathol. 2026 Aug 31;270(3):380–95. doi: 10.1002/path.70108 (PMC13635605; doi:10.1002/path.70108)
Supplement: Supplementary file 1 [file PATH-270-380-s001.docx]

**Comprehensive genomic profiling of mucosal melanoma reveals novel fusion transcripts and dysregulation of cell-cycle, MAPK, and PI3K pathways.**

M Monti *et al. J Pathol* <https://doi.org/10.1002/path.70108>

**Supplementary materials and methods**

**Supplementary Figures S1–S6**

**Supplementary Tables S1–S8**

Reference numbers refer to the main text list

**Supplementary materials and methods**

*Ethics approval and patient consent*

All human tissues used in this study were obtained under the approval of the Institutional Ethics Board of ASST Spedali Civili of Brescia (IRB code: NP 2066/2015) and ASST Sette Laghi of Varese (IRB code: NP 33025/2015), Italy, respectively. Informed consent was obtained from all patients for the use of human tissue and cell culture. Diagnosis of SN-MM was performed according to the available version of the World Health Organization Classification of Head and Neck Tumours and the American Joint Committee on Cancer/Union for International Cancer Control staging system, respectively [60].

*Cell culture*

Five human SN-MM cell lines, designated as SN-MM1 to SN-MM5, previously generated from tumour biopsies and extensively characterized by our group [21] were used in this study. Cutaneous melanoma (CM) cell lines (mel-336 and mel-327) were used as controls. The SN-MM and CM cell lines were cultured as previously described [21, 61]. Mycoplasma contamination was excluded by routine testing using the Universal Mycoplasma Detection Kit (Cat. No. 30-1012K; ATCC, Manassas, VA, USA) following the manufacturer’s protocol.

*Human tumour biopsies*

Formalin-fixed paraffin-embedded (FFPE) biopsies were retrieved from the archive of the Departments of Pathology of the ASST – Spedali Civili, Brescia, Italy and from Ospedale di Circolo e Fondazione Macchi, Varese, Italy. The series of patients partially overlaps that of a previous prognostic study on SN-MM from our groups [62].

*DNA isolation*

Genomic DNA was extracted from 2 × 10^6^ cells using the QIAamp DNA Blood Mini Kit (Cat. No. 51104; QIAGEN, Redwood City, CA, USA) following the manufacturer’s protocol. Automated DNA extraction was performed on the FFPE tissues using the Maxwell^®^ RSC DNA FFPE Kit (Cat. No. AS1450; Promega, Madison, WI, USA) following the manufacturer’s protocol.

*RNA isolation*

2.5 × 10^5^ cells were seeded in six-well plates in 2 ml of complete RPMI. Total RNA was isolated using the RNeasy Plus Micro Kit (Cat. No.74034; QIAGEN, Hilden, Germany). The homogenization step was performed by passing the lysate through a 20-gauge needle. In addition, the washing steps with Buffer RPE and 80% ethanol were repeated three and two times, respectively, to avoid guanidine salt contamination.

*Sanger sequencing*

PCR was performed using the specific primers reported in supplementary material, Table S1. The PCR products were purified using Amicon Ultra 0.5 ml Centrifugal Filters 30 K (Cat. No. UFC5030BK; Millipore, Burlington, MA, USA), following the manufacturer’s protocol, and sequenced in both directions using the BigDye™ Terminator v3.1 Cycle Sequencing Kit (Cat. No. 4337455; Applied Biosystems, Waltham, MA, USA). Excess fluorescent terminator dye was removed using Performa^®^ DTR Gel Filtration Cartridges (Cat. No. 4408228; Edge Bio Systems, San José, CA, USA). Samples were resuspended in formamide, denatured at 95 ℃ for 3 min, and processed through a SeqStudio™ Genetic Analyzer (Thermo Fisher Scientific, Waltham, MA, USA).

*RT-qPCR validation of fusion transcripts*

cDNA was synthesized by the qScriber™ cDNA Synthesis Kit (Cat. No. RTK0101; highQu GmbH, Kraichtal, Germany) from 150 ng of total RNA, in a total volume of 20 μl. Approximately 2 μl of the cDNA synthesis reaction was used for specific amplification of the target transcripts. RT-qPCR was performed in a total volume of 20 μl with 10 μl of SYBR Select Master Mix (Cat. No. 4472903; Life Technologies, Carlsbad, CA, USA) and 400 nm forward and reverse primers. Primers were designed using the appropriate fusion transcript sequence in Primer-BLAST (<https://www.ncbi.nlm.nih.gov/tools/primer-blast/index.cgi>?). Details on the primer sequences are reported in supplementary material, Table S2. All primers were manufactured by Metabion (Planegg, Germany). qPCR amplifications were carried out for 45 cycles using an annealing temperature of 58 °C. Melting curve analysis was performed to analyse the quality of the PCR products. RT-qPCR was performed using the ViiA 7 Real-Time PCR System (Applied Biosystems, Thermo Fisher Scientific). The results were analysed using StepOne v2.3 software (Applied Biosystems). The threshold cycle (Ct) was determined for each sample and relative expression levels were calculated using the ΔCt method and considered for statistical analysis. The *RPLP0* transcript was used for normalization. A negative control consisting of RNA from SN-MM cell lines expressing normal transcripts was included.

*Illumina TruSight Oncology 500* (*TSO500*) *HRD assay*

The SN-MM cell lines were analysed using the commercially available TruSight Oncology 500 HRD assay (Illumina, San Diego, CA, USA). The DNA analysis interrogated 523 genes for SNVs and InDels. In brief, DNA libraries were prepared following the manufacturer’s protocol, and DNA was fragmented using the Covaris^®^ ME220 Focused-ultrasonicator™ (Covaris, Woburn, MA, USA) to obtain fragments with an average length of approximately 150 bp. Sequencing was performed on the NextSeq 500 Sequencer (Illumina) using the NextSeq 500/550 High Output Kit v2.5 (300 cycles), following the manufacturer’s protocol. DNA variant filtering and annotation were performed using the DRAGEN TruSight Oncology 500 Analysis Software (v2.6.1). A custom variant filter was implemented using the OpenCravat tool (v2.11.1) to include only non-synonymous variants with a population allele frequency less than 1 × 10⁻⁴. Variants classified as variants of uncertain significance (VUS) or not available (NA) in the ClinVar database were also classified as pathogenic and Tier I or II according to the VarSome (v13.2.0) clinical database. Variants classified as benign or likely benign in the ClinVar database were excluded. Common single nucleotide polymorphisms (SNPs) were filtered out using the dbSNP (<https://www.ncbi.nlm.nih.gov/snp/>; last accessed 3 July 2025) and 1000 Genomes databases (<https://www.internationalgenome.org/home/>; last accessed 3 July 2025). The cut-off for variant allele frequency (VAF) was set at 5%. The TSO500 assay estimates TMB by calculating the number of eligible passing variants and dividing it by the size of the targeted coding region covered by the panel. A TMB value greater than 10 mutations per megabase (Mb) was classified as ‘high’ (≥10 mut/Mb).

*Whole exome sequencing* (*WES*)

DNA purity was measured using a Thermo Scientific™ NanoDrop Lite Spectrophotometer (Thermo Fisher Scientific, Waltham, MA, USA). DNA integrity was assessed using the TapeStation 4150 (Agilent Technologies, Santa Clara, CA, USA). All samples showed a DNA integrity number (DIN) greater than 7. DNA quantity was measured using the Qubit device and the Qubit BR DNA Kit (Thermo Fisher Scientific). Library preparation for Illumina sequencing was performed using Twist EF with the Universal Adapters protocol (Twist Bioscience, South San Francisco, CA, USA) following the manufacturer’s protocol, starting from 50 ng of DNA. Library size was assessed by capillary electrophoretic analysis using the Agilent 4150 TapeStation (Agilent Technologies) and quantity was measured using the Qubit BR assay and a Qubit device (Thermo Fisher Scientific). Exome enrichment was performed using the Twist target enrichment protocol (with the exome panel, version 1.3) (Twist Bioscience). Enriched libraries were quantified by real-time PCR using the KAPA Library Quantification Kit for Illumina platforms on a QuantStudio3 Real-Time PCR System (Thermo Fisher Scientific). WES libraries were pooled at equimolar concentration and sequenced on an Illumina NovaSeq 6000 (Illumina) using 150 bp reads in paired-end (PE) mode and generating 30 million fragments on average, resulting in 60–70× mapped coverage on the design of Twist Exome v1.3. FastQ files were quality controlled using the FastQC software (<http://www.bioinformatics.babraham.ac.uk/projects/fastqc/>). Reads were trimmed with FastP v.0.21.0 [63] to remove low-quality bases and sequencing adapters. Alignment of sequencing reads to the human reference genome (GRCh38/hg38) was performed with BWA-mem2 v2.2.1; then the SAM output was processed into a sorted BAM file with SAMTools v.1.13 [64]. The resulting BAM files were processed to remove duplicates and to perform base recalibration using Picard v.2.21.1 and GATK v.4.1.9.0 BaseRecalibrator (<https://gatk.broadinstitute.org/hc/en-us/articles/360036898312-BaseRecalibrator>), respectively. Clipping of overlapping reads was done by BamUtil v1.0.14 [65] and insert sizes’ calculation using CollectInsertSize by Picard v2.21.1 (<https://gatk.broadinstitute.org/hc/en-us/articles/360036856051-CollectHsMetrics-Picard>). Coverage and callable regions of the target were identified using the ‘coverage’ function by bedtools v.2.27.1 [66] and GATK CallableLoci v 3.8.1.0, while the on/near/off target and FOLD 80 penalty were calculated by applying the function ‘CollectHsMetrics’ by Picard v2.21.1. To detect both high- and low-frequency variants, the latter were called using GATK Mutect2 v.4.2.4.1 (<https://gatk.broadinstitute.org/hc/en-us/articles/360037593851-Mutect2>) and filtered for sequencing artefacts and contaminations using GATK FilterMutectCalls v4.2.4.1 (<https://gatk.broadinstitute.org/hc/en-us/articles/360036856831-FilterMutectCalls>). The resulting variants were annotated and prioritized using the VarSeq software (<https://www.goldenhelix.com/products/VarSeq/>). The default parameters were utilized when applying the different software.

*Gene variant annotation*

Functional annotation of the molecular alterations identified by next-generation sequencing (NGS) analysis was performed to assess whether the gene variants were associated with tumourigenicity and/or disease evolution, namely to describe their pathogenic effect (gain-of-function or loss-of-function mutations in oncogenes or tumour suppressor genes, respectively). To this aim, we referred to publicly available datasets for gene variant annotations, such as ClinVar v.2022_09_01, COSMIC v.92, TCGA v.2019_03_25, ICGC v.28, CIViC v.28, ExAC v.1.0, gnomAD 2.0.1 v2, 1000 Genomes, Phase3, ESP/EVS, ESP6500SI-V2, FGVD v.1.0.25, dbSNP v.155, RefSeq Genes v.110 NCBI, SIFT, Polyphen2, HVAR Pred, MutationTaster, MutationAssessor, FATHMM, FATHMM MKL, dbscSNV Spice Altering, 1.1, GWAS Catalog v.2017-07-31, and ExAC Functional Gene Constraints v.0.3.

We retained variants that were annotated as pathogenic/likely pathogenic in the ClinVar database or reported in the COSMIC database, with frequency of the alternate allele ≤ 1% (‘Rare Variants 1%’) or variant is missing in all the population frequency databases: 1000 Genomes Project, Exome Sequencing Project, ExAC (Exome Aggregation Consortium), gnomAD (Genome Aggregation), with damaging effect predicted by at least three tools out of five used (or missing), including Polyphen2, MutationTaster, MutationAssessor, FATHMM, SIFT, LoF or Missense variants, which also includes variants inside the splice regions, based on the RefSeq Genes database, and with frequency of the alternate allele ≤ 2% or missing in the Functional Genomics Variant Database (FGVD) comprising thousands of Italian individuals and centenarians.

*RNA sequencing*

RNA purity was measured using a NanoDrop Lite Spectrophotometer (Thermo Fisher Scientific) and RNA concentration was quantified using the Qubit RNA BR Assay Kit (Thermo Fisher Scientific). RNA integrity was assessed using the RNA 6000 Nano Kit on a Bioanalyzer (Agilent Technologies). All samples showed an RNA integrity number (RIN) greater than 8.

Directional RNA-seq library preparation was performed starting from 400 ng of total RNA using the TruSeq RNA Sample Prep Kit v2 (Illumina) after capturing poly-adenylated transcripts. RNA-seq libraries were size-selected (500–750 bp) on a BluePippin (Sage Science, Beverly, MA, USA). Library quality was assessed using a High Sensitivity DNA Kit on a 2200 TapeStation (Agilent Technologies, Wokingham, UK). Library size was assessed on a 2200 TapeStation (Agilent Technologies) and quantified by real-time PCR using the KAPA Library Quantification Kit (Kapa Biosystems, Wilmington, MA, USA). Sequencing was performed on an Illumina NovaSeq 6000 Sequencer (Illumina) generating ~33 million 150-bp paired-end reads per sample.

The quality of reads was assessed using FastQC software (<http://www.bioinformatics.babraham.ac.uk/projects/fastqc/>). Raw reads were trimmed with fastp (v.0.21.0) [9] with the --trim_poly_x parameter, to remove adapters and low-quality bases with default parameters. Filtered reads were aligned to the *Homo sapiens* reference genome (Ensembl version 109, RRID:SCR_002344) using STAR aligner (v2.7.9a) [67] with the parameter --peOverlapNbasesMin 5.

*Fusion transcript analysis*

Fusion transcripts of each cell line were identified on pooled RNA-sequencing replicates (~100 million fragments each). Fusion transcripts were identified with STAR-Fusion v1.10.0 [23] and Arriba v2.1.0 [24] using the default parameters. For STAR-Fusion, the impact of the fusion event on coding regions was predicted by setting the option --examine_coding_effect. Annotation using Arriba was conducted providing the software with a blacklist file comprising a list of recurrent alignment artifacts and fusion transcripts that can also be recognized in healthy tissue, a list of known fusions, and a file containing the protein domain annotation. The fusion allelic ratio (FAR) was calculated for each fusion transcript predicted by both software with FusionInspector v2.5.0 [68].

We retained fusions supported by at least five pieces of supporting evidence, namely five split-reads that overlap the fusion transcript junction or discordant read pairs where each mate maps to opposite sides of the chimeric junction. We also retained those fusions where the sum of the two types of reads was greater than 5. In addition, we removed low confidence variants and the one with a number of reads equal to 0. Fusion transcripts were annotated with FusionAnnotator [23] using the CTAT Human Fusion Lib database release v0.3.0. Subsequently, annoFuse R package v0.90.0 [69] was used to standardize output files and to further annotate fusions and filter them. Specifically, potential artefacts and fusion transcripts present in healthy tissues were filtered out if found in any of the following databases: GTEx_recurrent, DGD_PARALOGS, Normal, and BodyMap. Fusions comprising lowly expressed gene partners (FPKM < 1) were filtered out. Fusions between adjacent genes (read-through fusions) were excluded, except those reported in the Mitelman database (https://cgap.nci.nih.gov/cgap_mitelman_retire_notice.html).

Only in-frame fusion transcripts were considered for further analysis. Fusions with the breakpoint falling in the intragenic or intergenic region were removed. Fusion transcripts with a spanning DELTA (spanning reads count – junction reads count) greater than 10 were excluded. Fusions that were detected in FusionInspector and classified as ‘cosmic-like fusion’ or ‘cosmic-peak enriched’ were retained. The IGV program (version IGV_2.10.3) (https://software.broadinstitute.org/software/igv/) was used to inspect BAM files for read mapping on genes involved in fusion events.

To assess the 3D structural features of the studied fusion proteins, an *in silico* approach was employed. Specifically, AlphaFold3 (AF3) [25] was used to predict the folding of these fusion proteins based on amino acid sequences submitted to the prediction server as input. The output included the 3D model along with confidence metrics such as the predicted inter-residue distance distribution (pIDDT) and the predicted template modelling score (pTM), which collectively assess the reliability of the structural predictions (supplementary material, Table S3) [25]. Model visualization and image generation were performed using the UCSF Chimera molecular viewer [70].

To investigate the structural consequences of the MKRN1–BRAF fusion, the DeepSite tool [26] was employed to predict potential ligand-binding sites within the chimeric protein. This approach enabled the characterization of potential novel binding sites arising at the fusion interface, which were subsequently used for docking experiments with dabrafenib.

*Molecular docking analysis*

Structure-based computational tool provides insights into the binding pose of a ligand towards a macromolecular target along with an energy score, measured in kcal/mol, which indicates the calculated affinity between the species forming the complex and that considers the contributions of computed non-covalent interactions.

The initial structure used in this study was the crystallographic model of WT BRAF in complex with dabrafenib (PDB ID: 6V2U). In this structure, a portion of the protein corresponding to the so-called loop A is unresolved. To reconstruct the missing residues, the loop was modelled using the SuperLooper2 server [71]. Following loop completion, the dabrafenib docking was reproduced to validate the computational protocol. The docking was carried out with the AutoDock Vina algorithm [72], using a site-specific grid centred on the binding pocket to replicate the crystallographic pose of dabrafenib. The site-specific search grid parameters were set to centre *x* = −22.16, *y* = 4.27, *z* = 5.00, with a size of 16.29 × 12.31 × 14.09 Å. This provided the reference docking model of dabrafenib bound to BRAF WT. Regarding the V600E mutation, the PDB structure 4XV2 is available. Nevertheless, also in this case, the loop including residue 600 was not resolved. Thus, the same procedure was followed to obtain the V600E model for docking. Computational mutagenesis was applied in UCSF Chimera [70] to generate the mutant model corresponding to BRAF N581S. For this mutant, the docking of dabrafenib was repeated with the same grid parameters. Concerning vemurafenib, the crystallographic structure available in the PDB is 4RZV, which corresponds to BRAF in complex with vemurafenib in the R509H mutant form. Since no wild-type BRAF structure co-crystallized with vemurafenib is currently available, the R509H mutation was reverted to the wild-type sequence using UCSF Chimera [70], as described above. Subsequently, the N581S mutant model was generated through computational mutagenesis in the same environment. A blind docking strategy was applied in this case to avoid bias due to structural manipulation of the starting model. The search grid was centred at *x* = 87.15, *y* = 13.58, *z* = 18.80, with a size of 44.56 × 58.00 × 41.64 Å.

*Fluorescence* in situ *hybridization* (*FISH*)

FISH on interphase nuclei from paraffin-embedded 4-μm sections was performed using the ZytoLight SPEC BRAF break-apart probe (ZytoVysion, Bremerhaven, Germany). FISH was carried out following the manufacturer’s protocol. FISH images were captured at ×60 magnification and processed using the CytoVision DM5500B station. A minimum of 100 morphologically clear, non-overlapping nuclei from at least eight to ten areas were scored for each sample. Only experiments with at least 90% hybridization efficiency were considered. The tumour cells were considered to be FISH-positive when there were discrete green and red signals (>2 signal diameters) or individual red signals, while FISH-negative was defined as overlapping red and green signals (yellowish). Specimens were considered positive for specific gene rearrangement if more than 15% of the cells showed BRAF rearrangements were considered diagnostically positive when appear as ‘broken apart’ signals, red and green signals separated by a gap, or a ‘single orange (3′)’ signal (missing green signal), which indicates the loss of 5′ BRAF but preservation of the 3′ BRAF coding the kinase domain [73, 74]. A sample was considered positive if more than 15 of the accumulated 100 cells had discrete signals (≥15% split signals or single 3′ signals).

*MTS assay*

15 × 10^3^ cells per well were plated in 96-well plates in 125 µl of complete RPMI medium. Cells were treated with vemurafenib (10 mm; Cat. No. HY-12057; MedChemExpress, Monmouth Junction, NJ, USA) for 6 days and trametinib (10 mm; Cat. No. HY-10999, MedChemExpress) for 3 and 6 days. DMSO was used as a vehicle control at a final concentration of 0.15%. Cell viability was evaluated using the CellTiter 96^®^ AQueous One Solution Cell Proliferation Assay (Cat. No. G3582; Promega Corporation, Madison, WI, USA), following the manufacturer’s protocol. Absorbance was determined at 490 nm using the EnSight™ Multimode Plate Reader (PerkinElmer, Waltham, MA, USA). The sigmoidal dose-response inhibition function was applied to calculate the IC_50_ values.

*Western blotting*

1.5 × 10^5^ cells per well were seeded in 12-well plates in 1 ml of complete RPMI medium. The day after seeding, cells were starved in RPMI medium with 1% FBS for 24 h, then treated with 50 nm trametinib for 1 h. Cells were lysed in RIPA lysis buffer (Cat. No. 89900, Thermo Fisher Scientific) supplemented with Protease Inhibitor Cocktail (Cat. No. 78440; Sigma-Aldrich, St. Louis, MO, USA). Protein concentration was determined by the Bradford assay and 15 µg of total proteins was loaded onto 4–12% NuPAGE^®^ Bis-Tris Mini Gels (Cat. No. NP0335, Invitrogen) under reducing conditions and transferred onto a PVDF membrane (Cat. No. LC2007, Invitrogen). Membranes were blocked with 5% milk (Cat. No. 22012; Biotium, Fremont, CA, USA) in TBS-T (TBS with 0.05% Tween 20; Cat. No. 28360, Invitrogen) for 1 h at room temperature. Primary antibody pERK1/2 [Cat. No. 4376, clone 20G11, 1:1,000; Cell Signalling Technology (CST), Danvers, MA, USA] was incubated overnight at 4 °C in TBS-T with 5% BSA (Cat. No. A3059, Sigma-Aldrich); primary antibodies ERK1/2 (Cat. No. 4696, clone L43F12, 1:2,000; CST) and GAPDH (Cat. No. 5174, clone D16H11, 1:1,000; CST) were incubated overnight at 4 °C in T-TBS with 5% milk (Cat. No. 22012; Biotium, Fremont, CA, USA). The anti-rabbit (Cat. No. 31460, Thermo Fisher Scientific, RRID:AB_228341) or anti-mouse (Cat. No. 7076, CST, RRID:AB_330924) secondary antibodies conjugated with horseradish peroxidase were incubated for 1 h at room temperature. Detection was performed using the SuperSignal™ West Pico Chemiluminescent Substrate (Cat. No. 34577, Thermo Fisher Scientific) and visualized by autoradiography.

*Statistical analyses*

Statistical significance for *in vitro* experiments was determined using two-way ANOVA followed by the Bonferroni *post hoc* test for multiple comparisons. For all statistical tests, the level of significance was set at 0.05. Statistical analyses were performed using GraphPad Prism Software version 5 (GraphPad Software, Inc., La Jolla, CA, USA; RRID:SCR_002798).

**Figure S1.** **Validation of SNVs in SN-MM cell lines and corresponding parental tumours by Sanger sequencing.** Sequence chromatograph of genomic DNA from SN-MM cell line (upper panel) and parental tumour biopsies (lower panel) showing the SNVs, as labelled. The forward sequence is shown for *PTEN*. Reverse sequences are shown for *ABLIM3*, *PAH*, and *LRP1B*.

**Figure S2.** **Expression levels of mutated genes across SN‑MM cell lines. Box‑and‑whisker plots show TPM expression levels for genes harbouring SNVs or InDels across the five SN‑MM cell lines.** Genes displaying significantly higher or lower expression across cell lines are shown. The cell line harbouring the gene variant is reported in brackets and highlighted with bright colour. One-way ANOVA statistical analysis was performed, with adjusted *p* values (padj) reported.

**Figure S3.** **Expression of fusion transcripts and structural modelling of selected fusion proteins in SN‑MM.** (A) Expression of fusion transcripts in SN-MM cell lines. The mRNA relative expression (2^−ΔCt^) of each fusion transcript was measured by real-time RT-qPCR in the corresponding SN-MM cell line, as previously identified by RNA-seq. The mean and SD of biological replicates are shown (*n* = 3). (B) The CBFB–RUNX1 complex. The yellow region of CBFB, which contains the key interaction residue Asn104 (red) required for the binding of RUNX1 (cyan), represents the region that has been lost in the CBFB–ALG8 fusion protein. (C) CRLS1–MCM8 in complex with MCM9 (orange). The model was obtained by superimposing the predicted fusion protein with the MCM8–MCM9 dimer structure (PDB ID: 7WI7).

**Figure S4.** **Interphase FISH detection of BRAF rearrangement.** Representative images are shown for the FISH signal pattern of (A, B, D, E, G–K) BRAF rearrangement and (C, F) CEN7 in (A–C) the SN-MM2 cell line, (D–F) the SN-MM2 parental tumour biopsy, and (G–K) SN-MM cases of a retrospective cohort (*n* = 10). (A, D) Representative low-power views and (B, C, E, F) high-power views are shown. Representative images of (G–I) positive and (J, K) negative cases. (L) Diagram showing the BRAF break-apart probe design and genes involved in the target region.

**Figure S5.** **Molecular docking analysis of dabrafenib and BRAF variants.** (A, B) 3D model obtained by introduction of the missing loop in the 6V2U structure with SuperLooper 2 (beige) and N581S mutation (green). The mutated residue is depicted in red. Dabrafenib docked to WT protein is coloured in orange (−10.6 kcal/mol), while the one docked to the mutated protein is in blue (−8.4 kcal/mol). Docking grid encompassing the mutated residue is also shown. (C, D) Comparison between (C) the 4XV2 structure (namely V600E, but missing the loop) and (D) the one obtained with SuperLooper 2 and mutated with UCSF Chimera. Docking pose and score are only marginally affected by the mutation with respect to WT protein, as the residue is outside the docking grid. (E, F) 3D model obtained by introduction of the missing loop in the 4RZV structure with SuperLooper2, wild-type form (beige), and N581S mutation (green). The mutated residue is depicted in red. Vemurafenib docked to WT protein is coloured in purple (−11.8 kcal/mol), while the one docked to the mutated protein is in blue (−9.2 kcal/mol). Docking grid encompassing the mutated residue is also shown. (G–J) Comparison between the DeepSite analysis (purple) carried out on (G) BRAF (6V2U) and (H) the fusion transcript model. Residue V600, belonging to the loop coloured in yellow, is depicted in red. Docking analysis showed that (I) dabrafenib (blue) and (J) vemurafenib (purple) indeed preferentially bind to the new pocket identified by DeepSite.

**Figure S6. Full western blotting images corresponding to those shown in Figure 5.**

**Table S1.** List of PCR primers for genes evaluated by Sanger sequencing on SN-MM cell lines and corresponding parental tumours.

| **Gene** | **Sequence 5' to 3'** | ***T*_m_ (°C)** |
| --- | --- | --- |
| *ACOX3* F | GGGAAGGTAATACCTACCTC | 60 |
| *ACOX3* R | AGTGAGGTCCTTCTGGGAAT | 60 |
| *ABLIM3* F | TTTGCTCAGCAGTGTCCTCT | 60 |
| *ABLIM3* R | GGACCCACTTGCTGATATAC | 60 |
| *GP2* F | AACACCAGGTTAAACCGGGA | 60 |
| *GP2* R | GACGGGAATGGAGAGTTCAT | 60 |
| *KRT12* F | GTAGGACAGAAGACATGGTG | 60 |
| *KRT12* R | TGCCAGATCATTTCAGCCAG | 60 |
| *BRAF F* | TGCTTGCTCTGATAGGAAAATG | 58 |
| *BRAF R* | AGCATCTCAGGGCCAAAAAT | 58 |
| *ODF3L2 F* | GATCACTCCAAAGACTCTGC | 60 |
| *ODF3L2 R* | TCTTGGACCCCAAAGTCACA | 60 |
| *PAH F* | GACATTATCCAAGACAAACATG | 60 |
| *PAH R* | AAGCTATATTGAAGACAACTGC | 60 |
| *PRRT1 F* | ATGACTGCAGAAAGAGCCTG | 60 |
| *PRRT1 R* | TCTGTACCATCCTCACCGTA | 60 |
| *THBS1 F* | AACATGCTCAGCAGCCTGTT | 60 |
| *THBS1 R* | AGGGTTGGATGAAGTGGATC | 60 |
| *ATF7 F* | AGGGTTGGATGAAGTGGATC | 60 |
| *ATF7 R* | CTTTTCTCCAGGAGGTTACC | 60 |
| *PTEN F* | GTTTGATTGCTGCATATTTCAG | 60 |
| *PTEN R* | CAAAGCATTCTTACCTTACTAC | 60 |
| *TP53 F* | ATACGGCCAGGCATTGAAGT | 60 |
| *TP53 R* | TCATCTTCTGTCCCTTCCCA | 60 |
| *RYR1 F* | CCAAGAGGACTTCGTGCAG | 60 |
| *RYR1 R* | ATGAGCAGCGAGCGGATCT | 60 |
| *GSC2 F* | TCAGGATACTGGTTCTGCAC | 60 |
| *GSC2 R* | GTGCCCTTGTCTCTGGGTG | 62 |
| *NRAS F* | CAACAGGTTCTTGCTGGTGT | 58 |
| *NRAS R* | CCTCACCTCTATGGTGGGAT | 58 |
| *CYP21A2 F* | AAGAACTCCAGAGCTCTGG | 58 |
| *CYP21A2 R* | CATCTTGAGGATGACACTGC | 60 |
| *GK2 F* | CAGAGTATCCCTCTTGCACT | 60 |
| *GK2 R* | GAGACCTCAGGAGACATTGA | 60 |
| *IARS2 F* | TTGTTCTTGTTCAGGCTCTATT | 60 |
| *IARS2 R* | TCAGCCAGGTGAGGAAGAAT | 60 |
| *LRP1B F* | CAGTCTGTCCAGTAGACTTC | 60 |
| *LRP1B R* | CAGCCCTCTATGATGGAACA | 60 |
| *PRRG3 F* | GCTCTCTCCTCAGTGTTTCT | 60 |
| *PRRG3 R* | AGATCTCCTCCATGCACTCT | 60 |
| *CCDC66 F* | GTTGCGGTTTGTTAAAAATGAG | 60 |
| *CCDC66 R* | TGGAGACAATATTAGCTTGGTT | 60 |
| *ITIH5 F* | GCTGTCTGGAGTGACTGATA | 60 |
| *ITIH5 R* | CACAATTCTCCATGACCTCC | 60 |
| *KLK14* F | TGCACAGGTTGGTGTAGACA | 60 |
| *KLK14* R | TGTCTCCCTGTGTCTGCTTCT | 64 |
| *SCUBE2* F | TTGTACTGTGGATGGCAGCT | 60 |
| *SCUBE2* R | GCCCTGTGTTTGTGGAAATC | 60 |

**Table S2.** List of PCR primers for fusion transcripts evaluated by RT-qPCR.

| **Fusion transcript** | **Sequence 5' to 3'** | **Temperature (°C)** |
| --- | --- | --- |
| *CBFB–ALG8* F | GGTTTTTCTTTTCTTGCCTGC | 60 |
| *CBFB–ALG8* R | GGCCACAGGAACCAATCTGT | 62 |
| *MKRN1–BRAF* F | CAGCATATCAAAAAAACACTTGG | 62 |
| *MKRN1–BRAF* R | CTTTCCCTTGTAGACTGTTCC | 62 |
| *CCNB1–SCAMP1* F | GGTGAATGGGATCCATCAG | 58 |
| *CCNB1–SCAMP1* R | TGTGTATTGGGTACATTAGGCA | 62 |
| *CRLS1–MCM8* F | CTTATTCCAGTTTACAGCGA | 56 |
| *CRLS1–MCM8* R | CCACCTTCTGTCAGTTCTTTAA | 62 |
| *LDHB–C2CD5* F | GACTCAAGTGAACACCTGGA | 60 |
| *LDHB–C2CD5* R | GCTGCATCTGTCTGCAAAGG | 62 |
| *MBNL1–ITGA2* F | TCATTGAAAACATGTGGTCCTC | 62 |
| *MBNL1–ITGA2* R | AGTTGCAGGTGAGAAGCTGG | 62 |
| RPLP0 F | ATGCCCAGGGAAGACAGGGCG | 70 |
| RPLP0 R | CGAAGGGACATGCGGATCTGCTGC | 78 |

**Table S3.** Values of pTM score obtained for the studied models.

| **Fusion transcript** | ***MKRN1–BRAF*** | ***CBFB–ALG8*** | ***CCNB1–SCAMP1*** | ***LDHB–C2CD5* (isoform 1)** | ***LDHB–C2CD5* (isoform 3)** | ***CRLS1–MCM8*** | ***MBNL1–ITGA2*** |
| --- | --- | --- | --- | --- | --- | --- | --- |
| pTM score | 0.53 | 0.35 | 0.42 | 0.61 | 0.58 | 0.63 | 0.60 |

**Table S4.** SNVs and InDels of unknown clinical significance identified in SN-MM cell lines by using target NGS panel.

| **Cell line sample** | **Chr** | **Start position** | **Gene** | **Exon** | **HGVSc** | **HGVSp** | **Variant classification** | **VAF (%)** | **COSMIC** | **ClinVar** | **VarSome** |
| --- | --- | --- | --- | --- | --- | --- | --- | --- | --- | --- | --- |
| SN-MM1 | chr10 | 123298193 | *FGFR2** | 6 | c.661G>A | p.Val221Met | Missense mutation | 48 | NA | NA | Tier III |
| SN-MM1 | chr11 | 100999260 | *PGR** | 1 | c.542C>G | p.Ala181Gly | Missense mutation | 43 | NA | NA | Tier IV |
| SN-MM1 | chr22 | 38379410 | *SOX10* | 2 | c.382C>A | p.His128Asn | Missense mutation | 50 | NA | NA | Tier IV |
| SN-MM2 | chr1 | 120057096 | *HSD3B1** | 4 | c.950G>A | p.Arg317His | Missense mutation | 34 | NA | NA | Tier III |
| SN-MM2 | chr3 | 142272762 | *ATR* | 11 | c.2437A>G | p.Met813Val | Missense mutation | 100 | NA | VUS | Tier IV |
| SN-MM2 | chr5 | 1295161 | *TERT** | 5'UTR | c.-57A>C | NA |  | 15 | COSM1307007 | VUS | Tier III |
| SN-MM2 | chr6 | 26056217 | H1-2 | 1 | c.440C>T | p.Pro147Leu | Missense mutation | 57 | NA | NA | Tier IV |
| SN-MM2 | chr8 | 32621404 | *NRG1* | 9 | c.1587C>A | p.Ser529Arg | Missense mutation | 11 | NA | NA | Tier IV |
| SN-MM2 | chr13 | 48937017 | *RB1* | 8 | c.785G>A | p.Arg262Gln | Missense mutation | 70 | COSM1367190 | VUS | Tier IV |
| SN-MM2 | chr15 | 34649081 | *NUTM1** | 8 | c.2872C>T | p.Pro958Ser | Missense mutation | 39 | NA | NA | Tier IV |
| SN-MM2 | chr17 | 37682377 | *CDK12* | 13 | c.3568C>T | p.Pro1190Ser | Missense mutation | 66 | NA | NA | Tier IV |
| SN-MM2 | chr17 | 63533512 | *AXIN2** | 6 | c.1642G>A | p.Glu548Lys | Missense mutation | 30 | COSM5415294 | VUS | Tier IV |
| SN-MM2 | chr19 | 15297982 | *NOTCH3* | 11 | c.1774C>A | p.Arg592Ser | Missense mutation | 57 | COSM3283098 | VUS | Tier IV |
| SN-MM3 | chr1 | 120458310 | *NOTCH2* | 34 | c.7035G>A | p.Met2345Ile | Missense mutation | 35 | NA | NA | Tier IV |
| SN-MM3 | chr3 | 12653468 | *RAF1* | 3 | c.301C>T | p.Leu101Phe | Missense mutation | 22 | NA | NA | Tier III |
| SN-MM3 | chr3 | 38182669 | *MYD88* | 5 | c.783G>T | p.Lys261Asn | Missense mutation | 31 | NA | NA | Tier IV |
| SN-MM3 | chr4 | 55129854 | *PDGFRA** | 4 | c.388C>T | p.Pro130Ser | Missense mutation | 42 | COSM21975 | VUS | Tier III |
| SN-MM3 | chr4 | 55980328 | *KDR* | 6 | c.763G>C | p.Gly255Arg | Missense mutation | 46 | NA | NA | Tier IV |
| SN-MM3 | chr8 | 69069672 | *PREX2** | 36 | c.4346+4_4346+7del | NA | Splice site | 64 |  | NA | Tier IV |
| SN-MM3 | chr9 | 110249597 | *KLF4* | 3 | c.1078G>T | p.Val360Phe | Missense mutation | 31 |  | NA | Tier IV |
| SN-MM3 | chr10 | 123325171 | *FGFR2** | 3 | c.157G>T | p.Ala53Ser | Missense mutation | 8 |  | NA | Tier IV |
| SN-MM3 | chr16 | 89357134 | *ANKRD11* | 6 | c.500G>A | p.Arg167His | Missense mutation | 99 |  | VUS | Tier IV |
| SN-MM3 | chr19 | 45912211 | *POLR1G* | 3 | c.985A>G | p.Lys329Glu | Missense mutation | 20 |  | NA | Tier IV |
| SN-MM4 | chr1 | 150551412 | *MCL1* | 1 | c.595A>G | p.Met199Val | Missense mutation | 100 | NA | NA | Tier III |
| SN-MM4 | chr2 | 99189350 | *INPP4A* | 23 | c.2591G>A | p.Arg864Gln | Missense mutation | 24 | NA | NA | Tier IV |
| SN-MM4 | chr2 | 141625324 | *LRP1B** | 27 | c.4414C>T | p.Pro1472Ser | Missense mutation | 23 | COSM5910293 | NA | Tier III |
| SN-MM4 | chr2 | 141473672 | *LRP1B** | 37 | c.5894-1G>C | NA | Splice site | 34 |  | NA | Tier III |
| SN-MM4 | chr4 | 187525043 | *FAT1* | 19 | c.10637T>G | p.Leu3546Trp | Missense mutation | 70 | NA | NA | Tier IV |
| SN-MM4 | chr7 | 86468530 | GRM3* | 4 | c.1700A>T | p.Glu567Val | Missense mutation | 11 | . | NA | Tier III |
| SN-MM4 | chr13 | 110435204 | *IRS2* | 1 | c.3197C>T | p.Thr1066Ile | Missense mutation | 100 | NA | NA | Tier IV |
| SN-MM4 | chr16 | 72991833 | *ZFHX3* | 2 | c.2212A>C | p.Lys738Gln | Missense mutation | 64 | NA | NA | Tier III |
| SN-MM4 | chr17 | 37687481 | *CDK12* | 14 | c.4385C>A | p.Pro1462Gln | Missense mutation | 26 | . | NA | Tier IV |
| SN-MM4 | chr17 | 43362217 | *MAP3K14* | 6 | c.1252G>A | p.Glu418Lys | Missense mutation | 69 | NA | VUS | Tier IV |
| SN-MM4 | chr21 | 45649622 | *ICOSLG** | 6 | c.1213G>A | p.Gly405Arg | Missense mutation | 7 | . | NA | Tier IV |
| SN-MM4 | chr22 | 29130637 | *CHEK2* | 2 | c.73G>T | p.Val25Phe | Missense mutation | 20 | NA | NA | Tier IV |
| SN-MM5 | chr1 | 16246001 | *SPEN* | 8 | c.1624C>T | p.Pro542Ser | Missense mutation | 39 | NA | NA | Tier IV |
| SN-MM5 | chr2 | 61724121 | *XPO1* | 10 | c.781C>T | p.Arg261Ter | Nonsense mutation | 7 | NA | NA | Tier IV |
| SN-MM5 | chr8 | 41839376 | *KAT6A* | 4 | c.806G>A | p.Arg269Gln | Missense mutation | 69 | NA | NA | Tier IV |
| SN-MM5 | chr10 | 70405539 | *TET1** | 4 | c.3053_3055delinsGTG | p.Asn1018_Lys1019delinsSerGlu | Frameshift ins | 53 | . | NA | Tier III |
| SN-MM5 | chr13 | 110436315 | *IRS2* | 1 | c.2086G>T | p.Ala696Ser | Missense mutation | 21 | . | NA | Tier IV |
| SN-MM5 | chr16 | 10032027 | *GRIN2A** | 3 | c.796G>A | p.Glu266Lys | Missense mutation | 56 | NA | NA | Tier III |
| SN-MM5 | chr17 | 37883141 | *ERBB2* | 25 | c.3044G>A | p.Gly1015Glu | Missense mutation | 23 | NA | VUS | Tier IV |

*Genes with very low or absent expression (TPM < 5) across all samples.

ins, insertion.

**Table S5.** Fold change of the gene copy number variations in SN-MM cell lines.

| **Cell line →**  **↓ Gene** | **SN-MM1** | **SN-MM2** | **SN-MM3-P35** | **SN-MM3-P80** | **SN-MM4** | **SN-MM5** |
| --- | --- | --- | --- | --- | --- | --- |
| *MET* | 1.68848 | 2.19441 | 1.472 |  | 2.03306 | 18.837 |
| *CCND1* |  |  |  |  |  | 10.6798 |
| *FGF4* |  |  |  |  |  | 10.1805 |
| *FGF19* |  |  |  |  |  | 9.72938 |
| *FGF3* |  |  |  |  |  | 8.31922 |
| *CDK4* |  |  |  |  |  | 6.52752 |
| *RAC1* |  | 1.49236 | 1.53937 | 3.86834 | 2.65194 |  |
| *ETV1* |  | 1.70884 | 1.65953 | 3.82415 | 3.27217 |  |
| *NBN* | 2.4241 | 1.69083 | 2.32044 | 2.23411 | 3.58496 |  |
| *PREX2* | 2.33036 | 1.55285 | 1.95585 | 1.90415 | 3.33183 |  |
| *PMS2* |  | 1.58222 | 1.5083 | 3.22007 | 2.78027 |  |
| *RUNX1T1* | 2.23436 | 1.48552 | 1.83015 | 1.94798 | 3.20724 |  |
| *RPS6KB2* |  | 1.54038 |  |  |  | 3.17068 |
| *LYN* | 2.13313 | 1.55418 | 1.86585 | 1.84328 | 3.14417 |  |
| *PRKDC* | 2.17932 | 1.51866 | 1.89713 | 1.83822 | 3.04723 |  |
| *TCEB1* | 2.19951 | 1.52055 | 1.96517 | 1.80689 | 3.04716 |  |
| *INHBA* |  | 1.53378 | 1.51628 | 1.69148 | 3.03404 |  |
| *RAD21* | 2.21431 | 1.51852 | 1.84718 | 1.90112 | 3.0033 |  |
| *SOX17* | 2.03397 |  | 1.67871 | 1.74555 | 2.91437 |  |
| *MYC* | 2.10074 | 1.50435 | 1.75234 | 1.75534 | 2.88148 |  |
| *PIK3CG* |  | 2.10911 |  |  | 1.98687 | 2.79364 |
| *CARD11* |  |  |  | 2.7778 | 2.71193 |  |
| *RICTOR* |  | 2.64281 |  |  | 2.2618 |  |
| *MITF* |  |  |  |  |  | 2.59197 |
| *RECQL4* | 2.17344 | 1.62333 | 1.75098 | 1.59294 | 2.50659 |  |
| *AKT3* | 1.69643 | 1.70714 | 2.3256 | 2.46403 | 1.47207 |  |
| *MCL1* |  | 1.68301 | 2.17063 | 2.42164 |  |  |
| *ABL1* |  | 0.604651 |  |  | 2.39846 |  |
| *GLI1* |  |  |  |  |  | 2.36035 |
| *CDC73* | 1.61803 | 1.66702 | 2.21283 | 2.35583 | 1.45626 |  |
| *NAB2* |  |  |  |  |  | 2.31597 |
| *PTPN11* |  |  |  |  |  | 2.30424 |
| *NOTCH1* | 2.28412 |  |  |  | 1.65974 |  |
| *XRCC2* | 1.63217 | 2.26707 |  |  | 2.02351 |  |
| *NTRK1* | 1.4307 | 1.52838 | 1.87888 | 1.92528 |  | 2.18383 |
| *TBX3* |  |  |  |  |  | 2.14932 |
| *FGF10* |  |  |  |  | 2.14255 |  |
| *CUX1* |  | 2.14225 |  |  | 1.78818 |  |
| *RAF1* | 2.13564 |  | 1.50028 | 1.58898 |  |  |
| *MEF2B* | 2.11315 |  |  |  |  |  |
| *JAK3* | 2.1096 |  |  |  |  |  |
| *H3F3C* | 1.47219 |  |  | 2.10021 |  | 1.43855 |
| *IL10* |  | 1.58837 | 1.91524 | 2.08901 |  |  |
| *HGF* |  | 2.04808 |  |  | 2.08515 |  |
| *E2F3* |  |  | 2.05876 | 1.99243 | 1.81742 | 1.4375 |
| *KRAS* |  |  |  | 2.04673 |  |  |
| *RIT1* | 1.76391 | 1.53282 | 2.04475 | 2.0441 |  |  |
| *DDR2* |  | 1.42469 | 1.91041 | 2.03256 |  |  |
| *BRAF* | 1.95219 | 2.02897 |  |  | 1.86347 |  |
| *RHEB* |  | 2.02535 |  |  | 1.83469 |  |
| *ABL2* | 1.38995 | 1.44291 | 1.9167 | 2.0245 |  |  |
| *SMARCD1* | 1.393 |  |  |  |  | 2.01864 |
| *CDK6* |  | 2.0173 |  |  | 1.81913 | 1.78344 |
| *IRF4* |  |  | 2.0104 | 1.98494 | 1.68826 | 1.60474 |
| *EZH2* |  | 2.00931 |  |  | 1.88687 |  |
| *SMO* | 1.35463 | 2.00589 |  |  | 1.64923 |  |
| *MAGI2* |  | 2.00506 |  |  | 1.94689 |  |
| *IL7R* |  |  |  |  | 1.9918 |  |
| *SH2B3* |  |  |  |  |  | 1.98843 |
| *FH* |  |  | 1.98441 | 1.97467 |  |  |
| *PAX3* |  |  | 1.96966 | 1.94708 |  |  |
| *PIK3R2* | 1.96041 |  |  |  |  |  |
| *HSD3B1* |  |  |  |  | 1.94806 |  |
| *GRM3* |  | 1.94015 |  |  | 1.82565 |  |
| *ERCC2* | 1.93275 |  |  | 1.48308 |  |  |
| *EP300* |  |  | 1.91936 | 1.60431 |  |  |
| *CD79A* | 1.91778 |  |  |  |  |  |
| *MDM4* |  | 1.47761 | 1.88564 | 1.91688 |  |  |
| *CRKL* |  | 1.91567 |  |  |  |  |
| *RFWD2* |  | 1.45899 | 1.85933 | 1.91469 |  |  |
| *KLF4* | 1.91042 |  |  |  | 1.81106 |  |
| *ACVR1B* | 1.91012 |  |  |  |  |  |
| *AXL* | 1.90589 |  |  | 1.53679 |  |  |
| *BBC3* | 1.90388 |  |  | 1.50529 |  |  |
| *PAK7* |  |  | 1.70536 | 1.90381 |  | 1.5545 |
| *TGFBR1* | 1.89872 |  |  | 1.39323 | 1.84157 |  |
| *CDKN1A* | 1.88934 |  |  |  | 1.71169 | 1.823 |
| *TRAF2* | 1.88197 | 0.63686 |  |  | 1.52744 |  |
| *FANCL* |  |  | 1.57144 | 1.88125 |  |  |
| *HIST1H3B* |  |  |  |  | 1.87986 |  |
| *KEL* |  | 1.87982 |  | 0.586574 | 1.79196 |  |
| *SDHA* | 1.7761 |  |  |  | 1.87642 |  |
| *POLD1* | 1.87221 |  |  |  |  |  |
| *CIC* | 1.86804 |  |  | 1.44184 |  |  |
| *NTRK2* |  |  |  |  | 1.86456 |  |
| *SPTA1* |  |  | 1.78019 | 1.85398 |  |  |
| *H3F3B* | 1.84764 | 1.73185 |  |  |  |  |
| *HNRNPK* |  | 0.642101 |  |  | 1.84629 |  |
| *PARP1* |  | 1.45414 | 1.77421 | 1.84402 |  |  |
| *INPP4A* |  |  |  | 1.8352 |  |  |
| *HIST1H1C* |  |  |  | 1.83001 |  |  |
| *HIST1H2BD* |  |  | 1.48281 |  | 1.82509 |  |
| *HIST3H3* |  | 1.53457 | 1.80841 | 1.82422 |  |  |
| *MAP3K14* | 1.82353 |  |  |  |  |  |
| *IKBKE* |  | 1.4947 | 1.81589 | 1.70945 |  |  |
| *NF2* |  | 1.81577 | 1.39865 |  |  |  |
| *PTCH1* | 1.81439 | 0.628976 |  |  | 1.66261 |  |
| *EGFR* |  | 1.65619 | 1.50178 | 1.70474 | 1.80825 |  |
| *MAPK1* |  | 1.79774 |  |  |  |  |
| *ZNF217* |  |  | 1.56941 | 1.79664 |  |  |
| *PIM1* | 1.62441 |  |  |  | 1.77815 | 1.50922 |
| *LZTR1* |  | 1.77075 |  |  |  |  |
| *PPP2R1A* | 1.76809 |  |  |  |  |  |
| *GNAQ* |  |  |  |  | 1.75207 |  |
| *SYK* |  | 0.613842 |  |  | 1.75188 |  |
| *MLH1* | 1.74988 | 1.36977 |  |  |  |  |
| *ERCC1* | 1.74855 |  |  |  |  |  |
| *ACVR1* | 1.91012 |  | 1.73993 |  |  |  |
| *BLM* |  |  | 1.58391 | 1.73547 | 0.649996 |  |
| *EGFL7* | 1.73091 |  |  |  |  |  |
| *RAD51C* | 1.61516 |  | 1.72899 | 1.47996 |  | 1.45771 |
| *EWSR1* |  | 1.72875 |  |  |  |  |
| *REL* |  |  | 1.46548 | 1.728 |  |  |
| *IKZF1* |  |  |  |  | 1.72499 |  |
| *ASXL1* |  | 1.47326 | 1.49097 | 1.7233 |  | 1.68479 |
| *VEGFA* |  |  |  |  | 1.72069 | 1.70571 |
| *FANCG* | 1.71759 |  |  |  |  |  |
| *PPARG* |  |  | 1.5227 | 1.7165 |  |  |
| *PPP6C* |  | 0.626988 |  |  | 1.71328 |  |
| *NRG1* | 0.497806 | 0.506008 |  | 1.71028 |  |  |
| *TSC1* |  | 0.633552 |  |  | 1.70865 |  |
| *FGF7* |  |  | 1.70271 |  |  |  |
| *B2M* |  |  | 1.70071 |  | 0.621368 |  |
| *CCND3* |  |  |  |  | 1.70036 | 1.47947 |
| *NOTCH4* |  |  |  |  | 1.69478 | 1.59977 |
| *NOTCH3* |  |  | 1.56827 | 1.6895 |  |  |
| *DAXX* | 1.68709 |  |  |  | 1.63171 |  |
| *ETV6* | 1.68533 |  |  | 1.53458 |  | 1.63615 |
| *XPO1* |  |  | 1.50908 | 1.68084 |  |  |
| *FANCI* |  |  | 1.50944 | 1.6764 | 0.638681 |  |
| *PIK3C2B* |  | 1.46304 | 1.67135 | 1.67578 |  |  |
| *BCL2* |  |  | 1.48652 |  | 0.677608 | 1.66758 |
| *FANCC* | 1.65792 | 0.609305 |  |  | 1.57182 |  |
| *BAP1* |  | 1.65538 |  |  |  |  |
| *GID4* | 1.64202 |  |  |  |  |  |
| *ARFRP1* |  |  |  |  |  | 1.64118 |
| *AKT2* | 1.62927 |  |  |  |  |  |
| *FANCE* | 1.62693 |  |  |  | 1.61657 | 1.5514 |
| *CCNE1* | 1.62027 |  |  |  |  |  |
| *CHEK2* |  | 1.6087 |  |  |  |  |
| *SDHC* | 1.35121 | 1.38138 | 1.56966 | 1.60785 |  |  |
| *RAD51* |  |  | 1.60521 |  |  |  |
| *GNAS* |  |  |  | 1.60512 |  |  |
| *MDC1* | 1.41478 |  |  |  | 1.60118 | 1.40568 |
| *TSHR* |  |  | 1.56908 |  | 1.59877 |  |
| *DDX41* | 1.46981 |  |  |  |  | 1.59793 |
| *CSNK1A1* | 1.59719 |  |  |  | 1.49845 |  |
| *SF3B1* |  |  | 1.59676 |  |  |  |
| *AURKA* |  |  | 1.50852 | 1.59638 |  | 1.44327 |
| *PIK3R1* |  |  | 1.4807 | 1.59588 | 1.44177 |  |
| *MAP2K4* |  |  |  |  | 1.59344 |  |
| *YES1* |  |  | 1.59262 | 1.56642 | 1.58587 |  |
| *CSF1R* | 1.58909 |  |  |  | 1.50647 |  |
| *SRC* |  |  |  |  |  | 1.58727 |
| *CHD2* |  |  |  | 1.58707 | 0.581956 |  |
| *GABRA6* |  |  |  |  | 1.57922 |  |
| *IGF1* |  |  |  |  |  | 1.57786 |
| *PLK2* |  |  | 1.5393 | 1.57732 |  |  |
| *TOP1* |  |  |  | 1.57672 |  |  |
| *NTRK3* |  |  | 1.41427 | 1.57172 | 0.586807 |  |
| *RASA1* |  |  |  |  | 1.57114 |  |
| *LRP1B* |  |  | 1.56977 |  |  |  |
| *KEAP1* |  |  |  | 1.56798 |  |  |
| *GREM1* |  |  | 1.56394 |  | 0.599561 |  |
| *KLHL6* |  |  |  |  |  | 1.5626 |
| *PBRM1* |  | 1.55783 |  |  |  |  |
| *PIK3CA* | 1.46047 |  |  |  |  | 1.55268 |
| *FGFR1* |  | 0.528988 |  | 1.55175 |  |  |
| *BARD1* |  |  | 1.54756 |  |  |  |
| *PMS1* |  |  | 1.54702 | 1.38509 |  |  |
| *DNMT3B* |  |  | 1.444 | 1.50292 |  | 1.54467 |
| *KAT6A* | 0.460106 |  |  | 1.54223 |  |  |
| *GNA13* |  |  | 1.53972 |  |  |  |
| *PDK1* |  |  | 1.53572 |  |  |  |
| *CALR* |  |  | 1.53422 | 1.52758 |  |  |
| *IGF1R* |  |  |  | 1.5306 | 0.534189 |  |
| *PPP2R2A* | 0.430483 | 0.455316 |  | 1.52975 |  |  |
| *STAT4* |  |  | 1.52867 |  |  |  |
| *DCUN1D1* | 1.45458 |  |  |  |  | 1.52628 |
| *MSH3* |  |  |  |  | 1.52608 |  |
| *HOXB13* | 1.52561 |  |  |  |  |  |
| *STAG1* | 1.52095 |  |  |  |  |  |
| *GATA6* |  |  |  |  |  | 1.52047 |
| *MSH2* |  |  | 1.40137 | 1.52012 |  | 1.46467 |
| *EPHA3* | 1.5175 | 1.42136 |  |  |  |  |
| *PIK3C3* |  |  |  |  |  | 1.51555 |
| *RAD51B* |  |  | 1.51529 |  |  |  |
| *MAP3K1* |  |  |  |  | 1.51397 |  |
| *NKX3-1* | 0.408836 | 0.472336 |  | 1.50548 |  |  |
| *APC* | 1.49348 |  |  |  | 1.50052 | 1.43115 |
| *ERBB4* |  |  | 1.49975 |  |  |  |
| *SMAD3* |  |  | 1.40975 | 1.49849 | 0.397325 |  |
| *BTG1* |  |  |  |  |  | 1.49823 |
| *CASP8* |  |  | 1.4977 |  |  |  |
| *SOX2* |  |  |  |  |  | 1.49528 |
| *SMAD2* |  |  | 1.47049 | 1.49434 |  | 1.48723 |
| *SMAD4* |  |  |  |  |  | 1.4889 |
| *BCL2L11* |  |  | 1.48652 |  |  |  |
| *PTPRT* |  |  |  | 1.48137 |  |  |
| *MYD88* |  | 1.47595 |  |  |  |  |
| *MGA* |  |  | 1.47346 |  | 0.589877 |  |
| *ARID2* | 1.4694 |  |  |  |  |  |
| *PPM1D* | 1.41193 |  | 1.46928 |  |  |  |
| *DOT1L* |  |  |  | 1.46679 |  |  |
| *ALK* |  |  |  |  | 1.46581 |  |
| *BRIP1* | 1.402 |  | 1.46558 |  |  |  |
| *FRS2* | 1.46532 |  |  |  |  |  |
| *PDGFRB* | 1.45811 |  |  |  | 1.41705 |  |
| *DICER1* |  |  |  |  | 1.45774 |  |
| *MAP3K13* | 1.42657 |  |  |  |  | 1.45464 |
| *MST1R* |  | 1.44512 |  |  |  |  |
| *NCOR1* |  |  |  |  | 1.4435 |  |
| *PTPRS* |  |  |  | 1.44283 |  |  |
| *PIK3C2G* | 1.4306 |  |  |  |  | 1.44201 |
| *MALT1* |  |  |  |  | 0.629381 | 1.43592 |
| *FGF1* |  |  |  |  | 1.43343 |  |
| *CUL3* | 0.524989 |  |  | 1.43224 |  |  |
| *NCOA3* |  |  |  | 1.4298 |  |  |
| *TMEM127* |  |  | 1.42708 |  |  |  |
| *CTNNA1* | 1.42121 |  |  |  |  |  |
| *RHOA* |  | 1.41442 |  |  |  |  |
| *SPOP* | 1.41352 |  |  |  |  |  |
| *RAD52* | 1.37 |  |  |  |  | 1.41336 |
| *DNMT1* |  |  |  | 1.41251 |  |  |
| *PRKCI* |  |  |  |  |  | 1.40471 |
| *INHA* | 0.485687 |  | 1.39619 |  |  |  |
| *SMARCA4* |  |  |  | 1.39119 |  |  |
| *NOTCH2* |  |  |  | 1.37956 | 0.588464 |  |
| *RAD50* | 1.35805 |  |  |  | 1.37807 |  |
| *CTNNB1* |  | 1.34691 |  |  |  |  |
| *RANBP2* |  |  | 1.33124 |  |  |  |
| *TNFRSF14* |  |  |  | 0.721906 |  |  |
| *TRAF7* |  |  |  |  | 0.694078 |  |
| *JAK2* |  |  | 0.47746 | 0.503388 | 0.668029 |  |
| *SH2D1A* |  |  |  |  | 0.659517 |  |
| *ETV4* |  |  | 0.653224 | 0.656265 |  |  |
| *MYB* | 0.49944 | 0.655594 |  |  | 0.632061 |  |
| *ETV5* |  | 0.654755 | 0.644083 |  |  |  |
| *ANKRD11* |  |  | 0.623582 | 0.622286 | 0.654508 |  |
| *FGF8* |  |  | 0.633315 | 0.649199 |  |  |
| *ARID1B* | 0.433838 | 0.604322 | 0.647547 |  | 0.476511 | 0.649159 |
| *FGFR3* |  |  | 0.6478 | 0.609825 |  |  |
| *MAP3K4* | 0.437697 | 0.592062 | 0.647601 |  | 0.555127 | 0.623478 |
| *CD274* |  |  | 0.466093 | 0.487689 | 0.646471 |  |
| *PMAIP1* |  |  |  |  | 0.643379 |  |
| *ZBTB2* | 0.464144 | 0.637594 |  |  | 0.50806 |  |
| *FAM46C* |  |  |  |  | 0.636075 |  |
| *PRDM1* | 0.496908 | 0.631635 |  |  | 0.582231 |  |
| *FYN* | 0.45795 | 0.628827 |  |  | 0.595456 |  |
| *BCL6* |  | 0.628685 |  |  |  |  |
| *WISP3* | 0.477894 | 0.628519 |  |  | 0.609517 |  |
| *PARK2* | 0.505488 | 0.62552 |  |  | 0.604673 |  |
| *IFNGR1* | 0.427186 | 0.596863 |  |  | 0.590284 | 0.62505 |
| *GATA3* |  | 0.622661 |  |  |  |  |
| *ARID1A* |  |  |  |  |  | 0.621972 |
| *KIF5B* |  | 0.621896 |  |  |  |  |
| *ERCC5* |  |  |  |  | 0.618724 |  |
| *IGF2* |  |  |  |  |  | 0.612045 |
| *LATS1* | 0.515706 |  |  |  | 0.607398 |  |
| *EPHA7* | 0.512034 | 0.606449 |  |  |  |  |
| *PTPRD* |  |  | 0.374019 | 0.410538 | 0.601428 |  |
| *MLLT3* |  |  | 0.39461 | 0.44221 | 0.597682 |  |
| *QKI* | 0.467314 | 0.591638 |  |  | 0.563816 |  |
| *ZRSR2* |  |  |  |  | 0.588767 |  |
| *NRAS* |  |  |  |  | 0.584649 |  |
| *TNFAIP3* | 0.485901 |  |  |  | 0.584561 |  |
| *AMER1* |  |  |  |  | 0.582151 |  |
| *PDCD1* | 0.582009 |  |  |  |  |  |
| *FUBP1* |  |  |  |  | 0.580141 |  |
| *SPEN* |  |  |  |  |  | 0.579527 |
| *SDHB* |  |  |  |  |  | 0.577217 |
| *GATA1* |  |  |  |  | 0.576992 |  |
| *NUTM1* |  |  |  |  | 0.570043 |  |
| *RBM10* |  |  |  |  | 0.567361 |  |
| *PDCD1LG2* |  |  | 0.360383 | 0.416834 | 0.553899 |  |
| *BCL10* |  |  |  |  | 0.553201 |  |
| *BCOR* |  |  |  |  | 0.552927 |  |
| *ROS1* | 0.551301 |  |  |  |  |  |
| *ARAF* |  |  |  |  | 0.549078 |  |
| *MAP2K1* |  |  |  |  | 0.536303 |  |
| *JAK1* |  |  |  |  | 0.535768 |  |
| *IDH2* |  |  |  |  | 0.534433 |  |
| *KDM5C* |  |  |  |  | 0.531683 |  |
| *LAMP1* |  |  |  |  | 0.530674 |  |
| *GPR124* |  | 0.528996 |  |  |  |  |
| *ZNF703* | 0.460588 | 0.521977 |  |  |  |  |
| *IRS2* |  |  |  |  | 0.519923 |  |
| *CD276* |  |  |  |  | 0.518347 |  |
| *CDKN2B* | 0 | 0.45695 | 0.430912 | 0.501701 | 0.047359 |  |
| *PNRC1* | 0.498497 |  |  |  |  |  |
| *IRS1* | 0.483322 |  |  |  |  |  |
| *ESR1* | 0.470306 |  |  |  |  |  |
| *GATA4* | 0.454769 | 0.452869 |  |  |  |  |
| *CDKN2A* | 0.048232 | 0.036628 | 0.340936 | 0.383511 | 1.20E−05 |  |
| *NF1* |  |  |  |  | 0.342166 |  |

Gene copy number gains and losses are in red and blue, respectively.

**Table S6.** List of novel SNVs and InDels identified among the SN-MM cell lines by WES.

| **Cell line sample** | **Chr position** | **Gene** | **Exon** | **HGVSc** | **HGVSp** | **Variant classification** | **VAF (%)** | **COSMIC** | **ClinVar** | **VarSome** | **Parental tumour^†^** |
| --- | --- | --- | --- | --- | --- | --- | --- | --- | --- | --- | --- |
| SN-MM1 | 4:8414366 | *ACOX3* | 5 | c.469G>T | p.Ala157Ser | Missense mutation | 52 | COSM734458 |  | Tier IV | Yes |
| SN-MM1 | 5:149207040 | *ABLIM3* | 6 | c.481G>A | p.Gly161Ser | Missense mutation | 60 | COSM6591258 |  | Tier III | Yes |
| SN-MM1 | 16:20318345 | *GP2** | 7 | c.1093G>A | p.Glu365Lys | Missense mutation | 48 | COSM968005 |  | Tier IV | Yes |
| SN-MM1 | 17:40866188 | *KRT12** | 2 | c.617A>G | p.Asn206Ser | Missense mutation | 42 | COSM978976 |  | Tier IV | Yes |
| SN-MM2 | 6:32149232 | *PRRT1** | 4 | c.911G>T | p.Trp304Leu | Missense mutation | 24 | COSM7131970 |  | Tier IV | Yes |
| SN-MM2 | 12:102912816 | *PAH** | 2 | c.143T>C | p.Leu48Ser | Missense mutation | 58 |  | Pathogenic | Tier III | Yes |
| SN-MM2 | 15:39585503 | *THBS1* | 7 | c.1060C>T | p.Pro354Ser | Missense mutation | 53 |  | Likely pathogenic | Tier IV | Yes |
| SN-MM2 | 19:467664 | *ODF3L2** | 3 | c.334C>T | p.Arg112Trp | Missense mutation | 35 | COSM5617958 |  | Tier IV | Yes |
| SN-MM3 | 2:47803501 | *MSH6* | 5 | c.3261dupC | p.Phe1088Leufs*5 | Frameshift ins | 58 |  | Pathogenic | Tier III | NA |
| SN-MM3 | 9:12702411 | *TYRP1* | 5 | c.1057_1060delAACA | p.Asn353Valfs*31 | Frameshift del | 100 |  | Pathogenic | Tier III | NA |
| SN-MM3 | 12:53534607 | *ATF7*, *ATF7-NPFF** | 6, 6 | c.455G>A | p.Arg152His | Missense mutation | 84 | COSM3772664 |  | Tier IV | Yes |
| SN-MM3 | 19:38494495 | *RYR1* | 39 | c.6418C>T | p.Arg2140Trp | Missense mutation | 21 |  | Likely pathogenic | Tier III | Yes |
| SN-MM3 | 22:19149818 | *GSC2** | 2 | c.358C>T | p.Pro120Ser | Missense mutation | 60 | COSM132698 |  | Tier IV | Yes |
| SN-MM4 | 1:220141838 | *IARS2* | 20 | c.2450G>A | p.Arg817His | Missense mutation | 27 |  | Pathogenic | Tier III | Yes |
| SN-MM4 | 2:140867755 | *LRP1B** | 27 | c.4414C>T | p.Pro1472Ser | Missense mutation | 22 | COSM5910293 |  | Tier III | Yes |
| SN-MM4 | 4:79407119 | *GK2** | 1 | c.1082T>C | p.Val361Ala | Missense mutation | 12 | COSM6054025 |  | Tier IV | Yes |
| SN-MM4 | 6:32041006 | *CYP21A2** | 10 | c.1360C>T | p.Pro454Ser | Missense mutation | 27 |  | Likely pathogenic | Tier IV | Yes |
| SN-MM4 | 11:68939658 | *IGHMBP2* | 15 | c.2911_2912delAG | p.Arg971Glufs*4 | Frameshift del | 57 |  | Likely pathogenic | Tier III | NA |
| SN-MM4 | 17:16948979 | *TNFRSF13B** | 3 | c.204dupA | p.Leu69Thrfs*12 | Frameshift ins | 79 |  | Conflicting classifications | Tier III | NA |
| SN-MM4 | X:151700043 | *PRRG3** | 3 | c.55C>T | p.Arg19Cys | Missense mutation | 100 | COSM3913571 |  | Tier IV | Yes |
| SN-MM5 | 3:56558848 | *CCDC66* | 2 | c.14A>G | p.Asp5Gly | Missense mutation | 100 | COSM5974881 |  | Tier IV | Yes |
| SN-MM5 | 10:7585994 | *ITIH5* | 8 | c.1015C>T | p.Arg339Trp | Missense mutation | 42 | COSM3981138 |  | Tier IV | Yes |
| SN-MM5 | 11:9066793 | *SCUBE2** | 6 | c.664G>A | p.Gly222Ser | Missense mutation | 100 | COSM4829403 |  | Tier IV | Yes |
| SN-MM5 | 19:51078155 | *KLK14** | 6 | c.608A>G | p.Asp203Gly | Missense mutation | 48 | COSM3990202 |  | Tier IV | Yes |

*Genes with very low or absent expression (TPM < 5) across all samples.

^†^Validation of SNVs by using Sanger sequencing on the corresponding parental tumour biopsies.

ins, insertion; del, deletion; NA, not assessed.

**Table S7.** Gene expression level (TPM) by RNA-seq analysis across SN-MM cell lines.

| **HGNC_symbol** | **SN-MM1.1** | **SN-MM1.2** | **SN-MM1.3** | **SN-MM2.1** | **SN-MM2.2** | **SN-MM2.3** | **SN-MM3.1** | **SN-MM3.2** | **SN-MM3.3** | **SN-MM4.1** | **SN-MM4.2** | **SN-MM4.3** | **SN-MM5.1** | **SN-MM5.2** | **SN-MM5.3** |
| --- | --- | --- | --- | --- | --- | --- | --- | --- | --- | --- | --- | --- | --- | --- | --- |
| *ABLIM3* | 60.57 | 54.97 | 33.54 | 11.5 | 18.48 | 12.25 | 21.13 | 32.51 | 37.01 | 18.86 | 23.16 | 22.79 | 3.27 | 2.06 | 2.19 |
| *ACOX3* | 10.08 | 12.17 | 12.21 | 19.68 | 21.32 | 16.32 | 8.98 | 8.01 | 8.43 | 6.74 | 9.86 | 9.68 | 10.07 | 7.34 | 7.93 |
| *ANKRD11* | 55.92 | 51.27 | 31.39 | 51.93 | 55.37 | 59.49 | 34.73 | 37.06 | 50.55 | 26.22 | 37.26 | 43.77 | 34.77 | 43.83 | 41.28 |
| *ATF7* | 15.95 | 16.99 | 15.83 | 18.53 | 20.45 | 18.58 | 14.86 | 17.93 | 18.95 | 10.24 | 13.11 | 13.53 | 19.2 | 22.48 | 25.22 |
| *ATF7-NPFF* | 1.27 | 1.12 | 0.31 | 0.31 | 0.82 | 0.46 | 0.37 | 0.33 | 0.46 | 0.38 | 0.08 | 0.7 | 0.43 | 0.43 | 0.65 |
| *ATR* | 12.8 | 11.85 | 9.03 | 7.2 | 6.9 | 6.28 | 5.05 | 2.79 | 2.93 | 8.15 | 7.98 | 8.54 | 7.7 | 9.89 | 8.87 |
| *AXIN2* | 1.58 | 1.99 | 0.69 | 1.16 | 1.51 | 1.84 | 0.66 | 0.58 | 0.7 | 2.02 | 0.49 | 0.61 | 2.31 | 2.61 | 2.24 |
| *BRAF* | 16.35 | 14.62 | 14.34 | 21.54 | 25.87 | 22.94 | 4.11 | 4.73 | 5.74 | 6.55 | 11.93 | 11.3 | 7.01 | 8.94 | 7.57 |
| *CCDC66* | 12.57 | 10.93 | 5.9 | 6.92 | 8.83 | 6.5 | 8.22 | 4.77 | 6.49 | 8.18 | 8.24 | 10.59 | 4.88 | 8.36 | 7.21 |
| *CDK12* | 25.78 | 25.02 | 21.73 | 23.67 | 38.77 | 41.4 | 23 | 24.65 | 26.69 | 9.93 | 18.76 | 20.08 | 27.82 | 36.88 | 37.4 |
| *CHEK2* | 18.43 | 15.12 | 12.51 | 22.71 | 24.31 | 25.71 | 19.71 | 11.82 | 11.51 | 10.92 | 10.99 | 8.9 | 19.13 | 19.78 | 19.8 |
| *CYP21A2* | 0 | 0 | 0 | 0 | 0.17 | 0.03 | 0 | 0 | 0.1 | 0 | 0 | 0.04 | 0 | 0 | 0 |
| *ERBB2* | 40.85 | 37.79 | 32.83 | 22.31 | 23.27 | 23.64 | 20.46 | 11.86 | 15.23 | 26.46 | 21.37 | 19.34 | 24.87 | 24.91 | 25.26 |
| *FANCA* | 30.82 | 21.48 | 13.3 | 19.51 | 14.11 | 16.82 | 10.41 | 9.67 | 15.71 | 6.06 | 4.91 | 7.3 | 11.99 | 17.06 | 15.53 |
| *FAT1* | 211.01 | 128.44 | 185.32 | 40.02 | 104.97 | 75.81 | 48.61 | 60.31 | 65.84 | 54.07 | 83.7 | 82.46 | 26.17 | 46.23 | 48.18 |
| *FGFR2* | 5.96 | 4.8 | 2.06 | 0 | 0.25 | 0.09 | 3.57 | 2.62 | 2.79 | 0.5 | 0.24 | 0.13 | 0 | 0 | 0.07 |
| *GK2* | 0 | 0 | 0 | 0 | 0 | 0 | 0 | 0 | 0 | 0 | 0 | 0 | 0 | 0 | 0 |
| *GP2* | 0 | 0 | 0 | 0 | 0 | 0 | 0 | 0 | 0 | 0 | 0 | 0 | 0 | 0 | 0 |
| *GRIN2A* | 0.07 | 0.04 | 0.36 | 0.1 | 0 | 0.01 | 1.67 | 3.78 | 2.87 | 0.24 | 0.18 | 0.06 | 0.15 | 0.23 | 0.11 |
| *GRM3* | 0 | 0 | 0 | 0 | 0 | 0 | 0 | 0 | 0 | 0 | 0 | 0.01 | 0 | 0 | 0 |
| *GSC2* | 0 | 0 | 0 | 0 | 0 | 0 | 0.4 | 0.3 | 0.12 | 0.04 | 0.02 | 0 | 0.02 | 0 | 0 |
| *H1-2* | 100.94 | 95.53 | 102.59 | 39.2 | 35.66 | 28.27 | 38.82 | 55.35 | 47.1 | 317.72 | 377.04 | 375.37 | 77.54 | 74.16 | 73.56 |
| *HSD3B1* | 0 | 0 | 0 | 0 | 0 | 0 | 0 | 0 | 0 | 0 | 0 | 0 | 0 | 0 | 0 |
| *IARS2* | 100.79 | 109.27 | 99.84 | 121.7 | 115.99 | 116.43 | 100.5 | 79.3 | 83.42 | 54.21 | 61 | 53.68 | 112.33 | 115.19 | 116.3 |
| *ICOSLG* | 0.85 | 1.07 | 0.98 | 0.97 | 1.91 | 1.86 | 0.71 | 0.98 | 0.94 | 0.37 | 1.26 | 1.14 | 0.08 | 0.26 | 0.25 |
| *IGHMBP2* | 9.74 | 10.61 | 11.89 | 8.32 | 10.42 | 7.68 | 11.75 | 14.23 | 18.05 | 8.09 | 5.25 | 6.98 | 130.97 | 155.94 | 156.93 |
| *INPP4A* | 8.91 | 8.16 | 7.53 | 11.6 | 11.23 | 9.92 | 16.03 | 12.54 | 14.46 | 9.45 | 10 | 9.48 | 9.63 | 10.87 | 8.79 |
| *IRS2* | 12.1 | 17.02 | 15.17 | 8.12 | 10.33 | 7.73 | 17.53 | 21.86 | 24.92 | 2.46 | 2.85 | 2.91 | 10.08 | 17.06 | 15.07 |
| *ITIH5* | 14.09 | 16.26 | 28.17 | 140.57 | 120.98 | 155.99 | 24.54 | 31.74 | 27.05 | 16.26 | 9.92 | 9.68 | 0.16 | 0.12 | 0.28 |
| *KAT6A* | 9.34 | 8.87 | 7.48 | 16.28 | 18.43 | 19.06 | 27.78 | 32.31 | 36.45 | 9.05 | 12.1 | 12.38 | 13.98 | 19.25 | 16.87 |
| *KDR* | 49.77 | 47.1 | 46.07 | 7.08 | 12.61 | 18.25 | 0.43 | 0.34 | 0.55 | 0.62 | 0.49 | 0.69 | 3.66 | 3.9 | 4.5 |
| *KLF4* | 12.11 | 11.09 | 10.11 | 8.66 | 5.04 | 3.98 | 17.1 | 18.66 | 21.87 | 7.17 | 12.07 | 11.5 | 1.21 | 2.59 | 2.65 |
| *KLK14* | 0 | 0 | 0 | 0 | 0 | 0.1 | 0.3 | 0 | 0.1 | 4.28 | 3.49 | 3.78 | 0 | 0.08 | 0.04 |
| *KRT12* | 0.69 | 0.2 | 1.71 | 0.49 | 0.31 | 0.36 | 0 | 0.15 | 0.03 | 0 | 0 | 0 | 0.1 | 0.04 | 0.02 |
| *LRP1B* | 0 | 0 | 0.01 | 0 | 0.01 | 0.02 | 0.11 | 0.05 | 0.04 | 0.05 | 0.02 | 0.08 | 0.02 | 0 | 0.01 |
| *MAP3K14* | 11.7 | 11.4 | 7.28 | 4.3 | 12.26 | 10.79 | 15.4 | 23.57 | 26.17 | 18.87 | 49.79 | 48.04 | 1.73 | 3.58 | 4.51 |
| *MCL1* | 135.91 | 156.86 | 129.27 | 113.15 | 143.59 | 126.19 | 193.16 | 402.59 | 422.48 | 65.86 | 131.89 | 127.52 | 60.75 | 91.42 | 78.78 |
| *MSH6* | 40.12 | 41.17 | 41.35 | 34.95 | 37.11 | 43.34 | 22.77 | 18.11 | 20.27 | 32.68 | 38.75 | 34.42 | 52.97 | 52.83 | 56.4 |
| *MYD88* | 51.45 | 53.39 | 52.35 | 53.41 | 72.97 | 54.97 | 22.34 | 21.97 | 21.43 | 27.98 | 36.16 | 32.42 | 27.91 | 30.8 | 26.55 |
| *NF1* | 7.95 | 7.25 | 5.34 | 21.82 | 24.05 | 23.23 | 15.25 | 16.5 | 19.81 | 5.22 | 9.85 | 9.67 | 22.93 | 22.35 | 21.46 |
| *NOTCH2* | 31.09 | 26.45 | 24.86 | 25.42 | 35.15 | 27.47 | 38.05 | 48.1 | 42.6 | 13.47 | 17.1 | 15.91 | 25.25 | 36.93 | 29.15 |
| *NOTCH3* | 2.76 | 3.14 | 3.33 | 1.68 | 0.94 | 1.78 | 4.7 | 3.67 | 4.22 | 10.25 | 5.49 | 5.36 | 4.02 | 3 | 2.27 |
| *NRAS* | 32.99 | 45.61 | 42.13 | 21.57 | 33.21 | 26.77 | 51.2 | 75.87 | 68.32 | 8.2 | 17.37 | 14.21 | 47.3 | 59.15 | 48.44 |
| *NRG1* | 0.29 | 0.03 | 0.12 | 0.27 | 0.89 | 0.79 | 23 | 48.55 | 65.06 | 19.47 | 63.29 | 53.5 | 0.25 | 0.41 | 0.14 |
| *NUTM1* | 0 | 0 | 0 | 0 | 0.02 | 0.01 | 0 | 0 | 0 | 0 | 0 | 0 | 0 | 0 | 0 |
| *ODF3L2* | 0 | 0 | 0 | 0.05 | 0 | 0 | 0 | 0.05 | 0 | 0.17 | 0 | 0 | 0 | 0 | 0 |
| *PAH* | 0 | 0 | 0 | 0 | 0 | 0 | 0 | 0 | 0 | 0 | 0 | 0 | 0 | 0 | 0 |
| *PDGFRA* | 1.07 | 1.79 | 0.39 | 0.78 | 0.82 | 0.79 | 0.74 | 0.08 | 0.2 | 0.65 | 0.43 | 0.38 | 0.5 | 0.32 | 0.27 |
| *PGR* | 0.01 | 0 | 0 | 0 | 0 | 0 | 0 | 0 | 0 | 0.02 | 0.13 | 0.02 | 0 | 0 | 0 |
| *POLR1G* | 30.4 | 35.15 | 28.08 | 28.41 | 48.29 | 40.45 | 64.47 | 113.1 | 129.01 | 14.03 | 55.14 | 49.14 | 10.44 | 15.31 | 12.96 |
| *PREX2* | 0 | 0 | 0 | 0 | 0.02 | 0 | 0.01 | 0.13 | 0.03 | 0.08 | 0.02 | 0.33 | 0 | 0 | 0 |
| *PRRG3* | 0.03 | 0 | 0 | 0 | 0 | 0.83 | 0 | 0 | 0 | 0 | 0 | 0 | 0 | 0 | 0 |
| *PRRT1* | 0.7 | 0.39 | 0.42 | 1.81 | 1.63 | 1.4 | 2.03 | 0.97 | 1.54 | 2.62 | 1.41 | 1.27 | 1.67 | 0.97 | 1.03 |
| *PTEN* | 39 | 47.44 | 45.9 | 18.38 | 15.56 | 18.65 | 15.1 | 23.79 | 23.03 | 13.43 | 12.78 | 13.88 | 24 | 43.44 | 43.14 |
| *RAF1* | 191.86 | 191.48 | 137.34 | 63.21 | 73.77 | 75.26 | 112.55 | 100.05 | 121.86 | 41.12 | 53.69 | 57.91 | 70.74 | 73.69 | 75.77 |
| *RB1* | 22.46 | 26.92 | 25.88 | 17.77 | 19.46 | 15.75 | 12.82 | 14.94 | 14.75 | 9.33 | 12.24 | 9.76 | 31.52 | 29.1 | 26.27 |
| *RYR1* | 2.38 | 3.73 | 2.42 | 0.09 | 0.05 | 0.07 | 27.35 | 27.72 | 26.76 | 0 | 0 | 0 | 0.04 | 0.21 | 0.1 |
| *SCUBE2* | 3.86 | 2.6 | 4.04 | 3.41 | 4.08 | 4.41 | 0.05 | 0.11 | 0.2 | 0.21 | 0.19 | 0.43 | 4.09 | 3.54 | 3.53 |
| *SOX10* | 227.51 | 215.85 | 192.43 | 380.87 | 340.82 | 277.23 | 334.72 | 392.94 | 420.64 | 165 | 102.03 | 121.06 | 282.44 | 248.35 | 202.63 |
| *SPEN* | 26.73 | 23.06 | 12.08 | 13.96 | 20.22 | 21.77 | 21.22 | 28 | 50.69 | 13.77 | 19.31 | 20.79 | 7.56 | 11.38 | 12.11 |
| *TERT* | 0 | 0 | 0 | 0 | 0 | 0.02 | 0.03 | 0 | 0 | 0 | 0.04 | 0.01 | 0 | 0 | 0 |
| *TET1* | 0.41 | 0.32 | 0.27 | 0.43 | 0.22 | 0.32 | 0.75 | 0.43 | 0.43 | 0.42 | 0.23 | 0.24 | 1.19 | 0.9 | 0.75 |
| *THBS1* | 337.54 | 343.58 | 289.62 | 3.26 | 33.36 | 19 | 0 | 0.11 | 0.22 | 465.91 | 835.99 | 748.62 | 18.4 | 82.25 | 69.61 |
| *TNFRSF13B* | 0 | 0 | 0 | 0 | 0 | 0 | 0 | 0 | 0 | 0 | 0 | 0 | 0 | 0 | 0 |
| *TP53* | 30.37 | 22.68 | 21.74 | 23.36 | 19.27 | 13.91 | 98.68 | 84.37 | 69.76 | 111.99 | 150.52 | 125.5 | 20.69 | 21.7 | 19.71 |
| *TSC1* | 11.8 | 7.56 | 5.04 | 6.54 | 7.48 | 7.86 | 10.56 | 9.95 | 13.76 | 14.42 | 14.91 | 17.4 | 10.22 | 12.96 | 12.94 |
| *TYRP1* | 66.03 | 75.57 | 11.3 | 206.82 | 248.84 | 226.61 | 0 | 0.58 | 0.22 | 0.02 | 0 | 0 | 5,116.33 | 6,597.94 | 6,207.88 |
| *XPO1* | 115.76 | 133.85 | 99.88 | 95.25 | 123.41 | 105.7 | 211.48 | 166.59 | 169.75 | 78.56 | 97.1 | 85.19 | 127.31 | 156.24 | 151.3 |
| *ZFHX3* | 5.91 | 6.13 | 4 | 3.02 | 3.47 | 2.81 | 4.11 | 4.38 | 6.63 | 2.2 | 2.59 | 3.28 | 2.07 | 3.6 | 1.92 |

Experimental replicates are shown (*n* = 3). Genes harbouring SNVs or InDels classified as: Tier I/II according to the VarSome clinical database are highlighted in green; Tier III according to the VarSome clinical database are highlighted in orange; Tier IV according to the VarSome clinical database and VUS or pathogenic/likely pathogenic in the ClinVar database are highlighted in yellow; Tier IV according to the VarSome clinical database and NA in the ClinVar database are highlighted in white.

**Table S8.** Efficacy of MEKi and BRAFi treatment in SN-MM cell lines.

| **Drug** | **Sensitivity and efficacy** | **SN-MM1** | **SN-MM2** | **SN-MM3** | **SN-MM4** | **SN-MM5** | **Mel336** | **Mel327** |
| --- | --- | --- | --- | --- | --- | --- | --- | --- |
| Trametinib | IC_50_ nm (95% CI) | 2.2 | 1.9 | 3.4 | 59.6 | 47.5 | 0.9 | – |
|  | Efficacy % (mean ± SD) | 91 ± 4.4 | 78 ± 6.3 | 85 ± 9.4 | 55 ± 5.5 | 70 ± 6.6 | 88 ± 7.9 | – |
| Vemurafenib | IC_50_ µm | 4.6 | 13.5 | 15.2 | NR | NR | 0.4 | NR |
|  | Efficacy % (mean ± SD) | 91 ± 2.3 | 65 ± 9.2 | 57 ± 7.5 | 14 ± 7.5 | 32 ± 7 | 98 ± 2.6 | 4 ± 22.5 |

NR, not responsive.
